# Antiproliferative Activity, Proapoptotic Effect, and Cell Cycle Arrest in Human Cancer Cells of Some Marine Natural Product Extract

**DOI:** 10.1155/2020/7948705

**Published:** 2020-11-25

**Authors:** Hong Cui, Mansour A. E. Bashar, Islam Rady, Hussein A. El-Naggar, Lamiaa M. Abd El-Maoula, Ahmed B. M. Mehany

**Affiliations:** ^1^Department of Hepatobiliary and Pancreatic Surgery, Tumor Hospital of Zhengzhou University, Zhengzhou, China; ^2^Zoology Department, Faculty of Science, Al-Azhar University, Cairo, Egypt; ^3^Masonic Cancer Center, University of Minnesota, Minnesota, USA; ^4^Nutrition and Food Science Department, Faculty of Home Economics, Al-Azhar University, Egypt

## Abstract

Bioactive constituents of numerous marine organisms have been investigated recently for their preclinical and clinical anticancer activity. Three marine organisms: black-spotted sea cucumber: *Pearsonothuria graeffei* (*Pg*), lollyfish: *Holothuria atra* (*Ha*), and sea hare: *Aplysia dactylomela* (*Ad*), were collected during winter 2019 from Gulf of Aqaba, Red Sea, Egypt, and macerated with ethanol into three different extracts: ***Pg*E**, ***Ha*E**, and ***Ad*E**, where each was *in vitro* assessed for its antiproliferative and proapoptotic properties on **HepG2**, **HCT-116**, and **MCF-7** cancer cells. ***Pg*E** dose-dependently inhibited the growth of **HepG2**, **HCT-116**, and **MCF-7** cells within IC_50_ values 16.22, 13.34, and 18.09 *μ*g/mL, respectively, while the IC_50_ values for the antiproliferative activity of ***Ha*E** were 12.48, 10.45, and 10.36 *μ*g/mL, respectively, and the IC_50_ values of ***Ad*E** were 6.51, 5.33, and 6.87 *μ*g/mL, respectively. All extracts were found to induce G_0_/G_1_ cell cycle arrest for **HepG2** cells side by side with their inhibition of **CDK2** on all three cell lines while all extracts were also showed to induce apoptosis in **HepG2** cell line at pre-**G**_**1**_ phase supplemented by their anticancer activity via proapoptotic protein **Bax**, **caspase-3**, and cleavage **PARP** increase, and antiapoptotic protein **Bcl-2** downturn. Moreover, necrosis has been relatively noticed in **HepG2** cell line as an additional anticancer activity for each extract. Our data introduced three ethanolic marine extracts as natural chemotherapeutic agents to be further developed for cancer control.

## 1. Introduction

Cancer remains the second causative agent of deaths worldwide [1, 2]. Annually, there are more than 10 million new cancer patients with over 6 million correlated deaths accounting for roughly 12% of worldwide mortality [3]. Therefore, there is an urgent need to determine the way of treatment of this ailment where the current strategy in treatment are mainly radiation-based therapy and chemotherapy [4]. However, this strategy is associated with toxic effects such as causing hair loss and other serious adverse effects [5]. Nowadays, the utilization of complementary and alternative medicines is more popular as a promising strategy for cancer therapy [3]. Natural products have been studied in a variety of models of cancer where the results have been extremely promising [4].

Water bodies are considered a source rich in bioactive compounds which add suitability progress and serve as a chemical shield against and sometimes protect from other creatures. These bioactive compounds have biological properties such as antioxidant, antibacterial, and antitumor [6, 7]. Marine fauna such as sponges, mollusks, echinoderms, ascidians, and corals are taxonomically different and have a variety of pharmacological activities that give us a tremendous research opportunity attraction for the discovery of novel anticancer therapeutics [8]. Researchers have been looking for marine derivative products 50 years ago, whereas in the last few decades, about 3000 new compounds have been isolated from various marine sources and assessed for their anticancer efficacy [9]. In recent years, many marine anticancer bioactive compounds have been isolated, characterized, identified, and preclinically assessed, and they are under clinical trials for human use [7]. Recently, marine natural products are considered the most interesting source of bioactive compounds and therapeutic agents [7]. During the last decades, marine sources are chemically mediated so-called secondary metabolites or natural products [10–12]. In the beginning, secondary metabolites have been identified as metabolic waste products or remains of primary metabolic overflow without any obvious biological function, but now, there is no doubt that these substances are playing a fundamental role in ecology and reproduction [13], while primary metabolites such as carbohydrates, amino acids, and fatty acids are chemically almost found in most organisms differ from secondary metabolites which are higher in chemical diversity associated with limited phylogenetic distribution, whereas the major structural classes of natural products include alkaloids, terpenes, polyketides, peptides, and shikimate-derived metabolites [14]. Marine chemical ecology has been known as a kind of young field due to the recent investigations in the last 25 years for the natural compounds mediating interactions between marine invertebrates compared to natural products isolated from terrestrial plants and microorganisms since the 1930s, and this is partly due to the recent development of underwater technology and new establishment of the ecologically relevant bioassays [15]. Besides the nutritional values of sea cucumber, there are therapeutic medicinal benefits that can be linked to the presence of bioactive compounds which generated a lot of unique biological and pharmacological activities such as antiangiogenic, anticoagulant, antihypertension, anti-inflammatory, antimicrobial, antioxidant, antithrombotic, antitumor, and wound healing [16–18]. A lot of bioactive compounds have been extracted, characterized, and purified from many animals, for instance, tunicates, sponges, soft corals, bryozoans, cephalopods, and echinoderms [19–22]. Also, among marine organisms, there are important commercial groups such as marine holothurians which are spiny skinned echinoderm invertebrates [23]. Therefore, it is possible to isolate a lot of bioactive compounds from marine fauna that may contribute to better health and serve as novel medication [24]. Here, an *in vitro* evaluation of the anticancer effects of three marine invertebrate crude extracts is presented.

## 2. Results

### 2.1. Antiproliferative Activity

**Figure 1:**
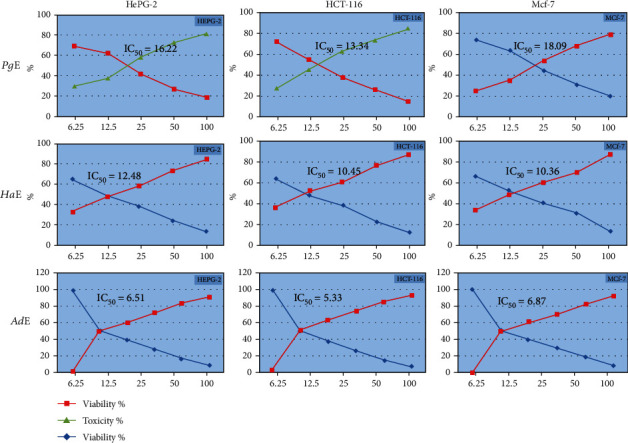
Antiproliferative activity of ***Pg*E**, ***Ha*E**, and ***Ad*E** against **HePG2**, **HCT-116**, and **MCF-7** cell lines.

**Figure 2:**
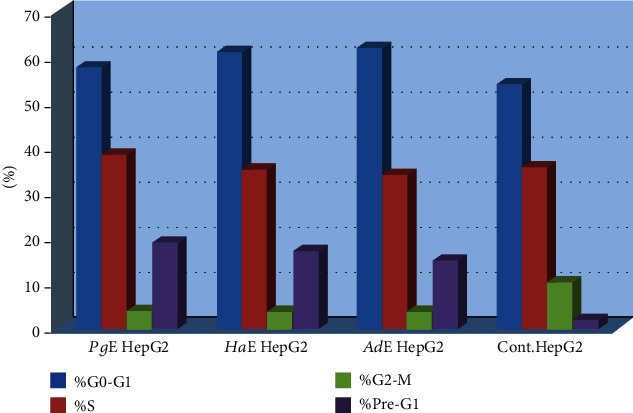
Cell cycle analysis effect in **HepG2** cells treated with ***Pg*E**, ***Ha*E**, and ***Ad*E**.

**Figure 3:**
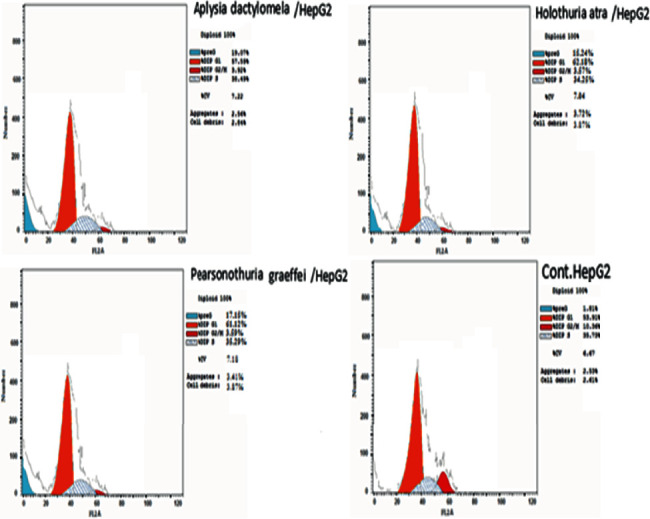
Cell cycle analysis and apoptosis effect in **HepG2** cell line treated with ***Pg*E**, ***Ha*E**, and ***Ad*E**.

**Figure 4:**
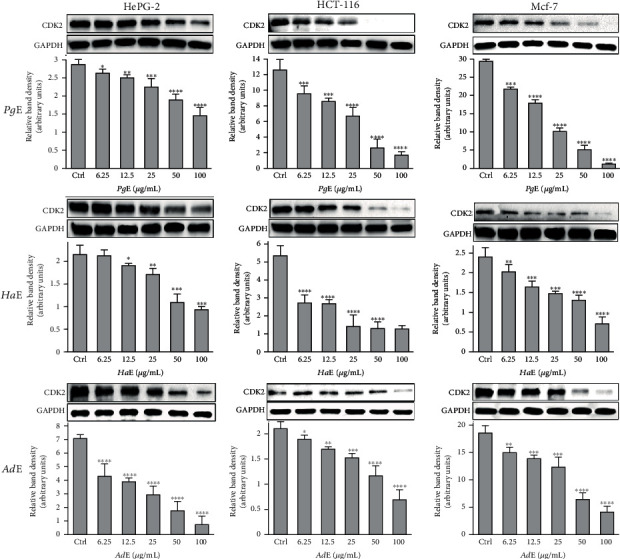
Modulation of CDK2 protein expression in **HePG2**, **HCT-116**, and **MCF-7** cells was treated dose-dependently with ***Pg*E**, ***Ha*E**, and ***Ad*E** and harvested 24 h after treatments. The immunoblots shown are representative of three independent experiments which all gave similar results where only representative result was cropped and inserted here. Bars represent the means ± SD. Single, double, triple, and quadruple asterisks are used for *p* < 0.05, *p* < 0.01, *p* < 0.001, and *p* < 0.0001, respectively, vs. control (DMSO-treated) cells.

All extracts inhibited the cell growth, each in all three cell lines in a dose-dependent manner with a remarkable cell viability decrease associated with cytotoxicity increase (Table 1 and Figure 1). The IC_50_ values for ***Pg*E**, ***Ha*E**, and ***Ad*E** on **HePG2** were 16.22, 12.48, and 6.51 *μ*g/mL, respectively, while on **HCT-116** were 13.34, 10.45, and 5.33 *μ*g/mL, respectively, whereas on **MCF-7** were 18.09, 10.36, and 6.87 *μ*g/mL, respectively. Side by side, the **HepG2** cell line was selected to study the cell cycle analysis (Figures 2 and 3). **HepG2** cell accumulation was noticed at the G0/G1 phase; the percentages were 57.59, 61.12, and 62.18% upon treating cells with ***Pg*E**, ***Ha*E**, and ***Ad*E**, respectively. This cell cycle analysis outcome is supported by a significant inhibition of cell cycle regulatory protein **CDK2** (Figure 4).

### 2.2. Apoptosis and Necrosis

**Figure 5:**
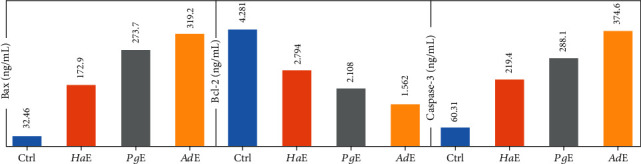
Comparative modulation of **Bax**, **Bcl-2**, and **caspase-3** protein expressions in **HePG2** cells was treated with ***Pg*E**, ***Ha*E**, and ***Ad*E** and harvested 24 h after treatments.

**Figure 6:**
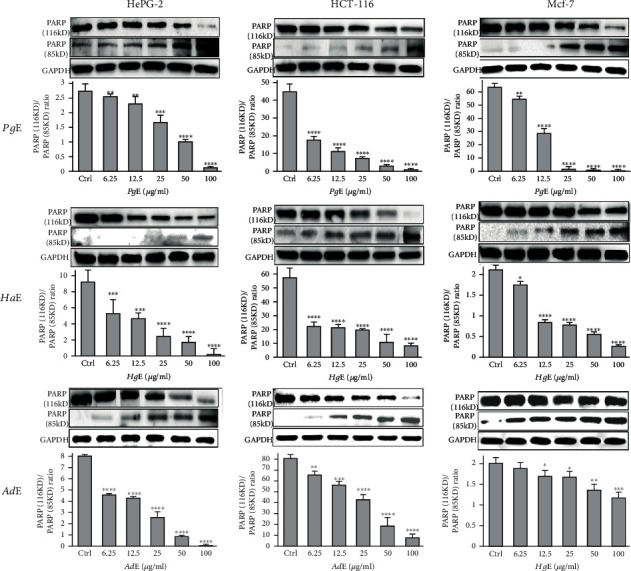
Modulation of the apoptotic protein biomarkers: **PARP** (116 kD) and cleaved **PARP** (85 kD) in **HePG2**, **HCT-116**, and **MCF-7** cells, was treated dose-dependently with ***Pg*E**, ***Ha*E**, and ***Ad*E** and harvested 24 h after treatments. The immunoblots shown are representative of three independent experiments which all gave similar results where the only representative result was cropped and inserted here. Bars represent the means ± SD. Single, double, triple, and quadruple asterisks are used for *p* < 0.05, *p* < 0.01, *p* < 0.001, and *p* < 0.0001, respectively, vs. control (DMSO-treated) cells.

**Figure 7:**
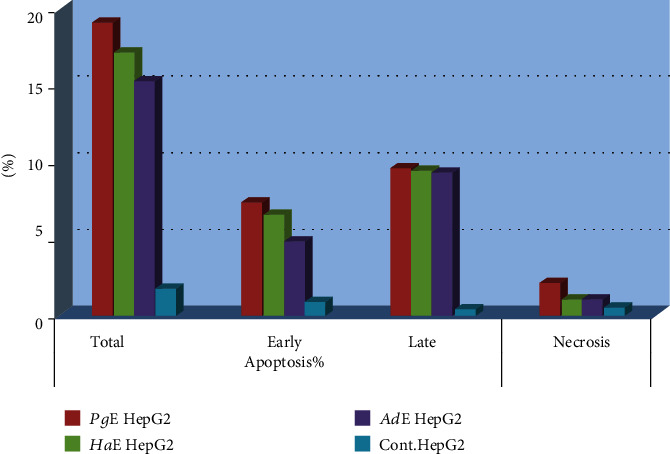
Percentage of apoptosis and necrosis induced by ***Pg*E**, ***Ha*E**, and ***Ad*E** in **HepG2** cells.

**Figure 8:**
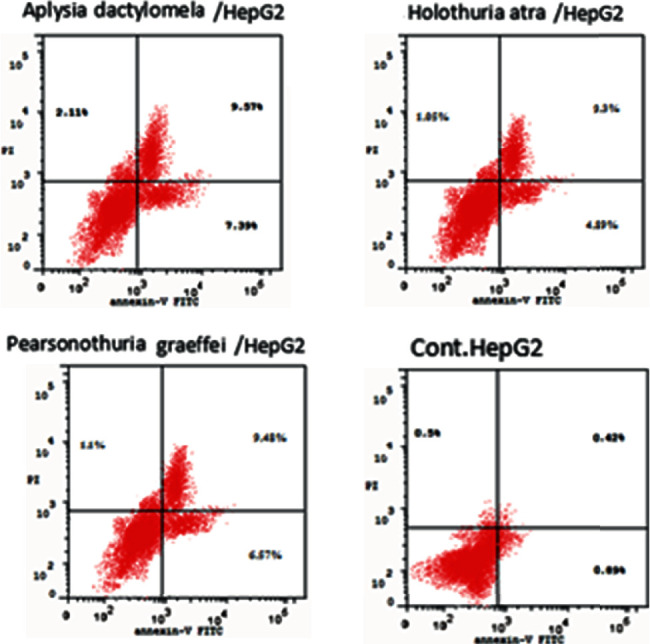
Cell death percentage induced by ***Pg*E**, ***Ha*E**, and ***Ad*E** in **HepG2** cells.

Each extract of *Pg*, *Ha*, or *Ad* induced apoptosis in **HepG2** cell line at the **pre-G1** phase (Figure 4). In addition, each extract increased the level of the proapoptotic protein **Bax** and **caspase-3** and decreased the level of the antiapoptotic protein Bcl-2 on HepG2 cancer cell line (Figure 5). Also, each extract induced apoptosis via cleavage **PARP** increase in **HepG2**, **HCT-116**, and **MCF-7** cancer cells (Figure 6). Annexin V/propidium iodide double staining (PI) assay was also used to investigate the mode of induced **HepG2** cell death caused by each extract treatment (Figure 7). The total percent of cell death is represented in Figure 8. So, necrosis has been relatively noticed in the percentage of cell death that has caused in **HepG2** cell line as an additional anticancer activity for each extract.

**Figure 9:**
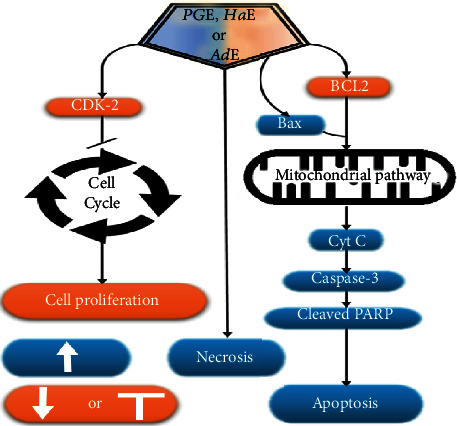
Schematic drawing of the mechanism of action of ***PG*E**, ***Ha*E**, and ***Ad*E** on **HepG2** cancer cells. This cartoon is based on the currently available data throughout the present study.

In summary, it seems that ***Pg*E**, ***Ha*E**, or ***Ad*E** each produced marked induction apoptosis and antiproliferative activity on **HepG2**, **HCT-116**, and **MCF-7** cancer cells via multiple pathways (Figure 9).

## 3. Material and Methods

### 3.1. Sampling and Identification of Marine Specimens

The marine organisms were collected via scuba diving from different sites at the Gulf of Aqaba, Egypt, during winter 2019. The taxonomy details were studied, and the voucher specimen was deposited at Marine Laboratory, Department of Zoology, Faculty of Science, Al-Azhar University, Egypt, with a registration number of MZ1005. Identification of samples was carried out according to Ruggiero et al. [25] as well as consultation of marine scientists through the World Wide Web as a confirmation for the present identification.

### 3.2. Identification and Description of the Collected Specimens

**Figure 10:**
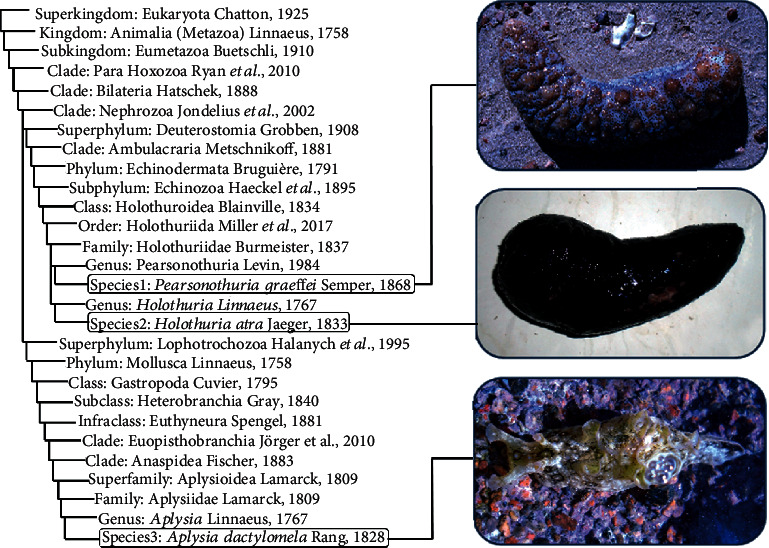
Taxonomical positions and photographs of *Pg*, *Ha*, and *Ad*.

The collected marine specimens were identified according to Ruggiero et al. [25] as two echinoderms: black-spotted sea cucumber: *Pearsonothuria graeffei* (*Pg*) and lollyfish: *Holothuria atra* (*Ha*), and one molluscan species *Aplysia dactylomela* (*Ad*) (Figure 10).

#### 3.2.1. *Pearsonothuria graeffei* (Semper, 1868)

*(1) Ecology*. Black-spotted sea cucumber is a coral reef species rarely found in depths of more than 25 m, mostly found associated with reef. It is tropically distributed, mostly found on reef slopes close to the coast, abundant on corals mixed with calcareous red algae.

*(2) Description*. It has a roughly cylindrical (Figure 10), thin-walled body (0.4 cm) that grows to about 30 cm (12 in) in length. Its body is arched dorsally (bivium) and slightly flattened ventrally (trivium). Trivium is grey, also with small black spots. It has white conical papillae sparsely distributed on bivium and long and large podia on trivium. Mouth ventrally, at one end, is surrounded by a ring of up to 24 leaf-like, paddle-shaped tentacles with black stalks which are black on the upper side and white beneath. 25 large, black-brown dots and numerous small black spots are surrounding the mouth. The anus is at the other end of the body, and there are several rows along the underside. The color of the adults is pale brown and white, with black speckles and small thorn-like protuberances. Its mean live weight is 700 to 1300 g.

#### 3.2.2. *Holothuria (Halodeima) atra* Jaeger, 1833

*(1) Ecology*. Lollyfish is a common shallow-water benthic species, rarely found at depths of more than 20 m, mostly on inner and outer reef flats and back reefs or shallow coastal lagoons; they are abundant on sandy-muddy grounds with rubble or coral patches and in seagrass beds.

*(2) Description*. It has a cylindrical (Figure 1), elongated body with rounded ends. It can grow to a length of 60 cm (24 in), but 20 cm (7.9 in) is a more common size (body width: 10 cm and body wall thickness: 0.8 cm). They have a tegument smooth, pliable, entirely black skin which is often covered by sand but also showing round patches lacking sand. The mouth is ventral on the underside at one end and is surrounded by a fringe of 20 black, branched tentacles. The anus is terminal at the other end. Red toxic fluid is secreted upon rubbing the body surface vigorously. Podia on bivium are sparsely distributed, ending in a small disc around 150 micrometers in diameter; podia on trivium are numerous, short, and stout. The mean live weight is about 200 g to 1000 g.

#### 3.2.3. *Aplysia dactylomela* Rang, 1828

*(1) Ecology*. The spotted sea hare is worldwide, being found in almost all and warm waters. It is commonly found in shallow waters, tide pools, rocky and sandy substrates; it also will be found feeding in beds. During the day, it will mostly hide under large rocks and in crevices. It can be found in a depth range from 0 to 20 m. It is capable of swimming and crawling. It squirts purple ink if it is disturbed; this ink is an irritant that causes “altered behaviour” in other invertebrates and fish. It contains leathery skin which make it practically inedible to most predators.

*(2) Description*. A beautifully patterned sea hare grows up to 40 cm long (Figure 10), with body shades of green, brown, and cream and a surface with black rings of varying sizes connected by a network of black lines and punctuated by cream spots. The foot is broad and well developed, its anterior end is rounded, and the posterior end is more bluntly pointed. The foot has a rough texture, in contrast to the smooth soft surface of the rest of its body. The parapodia are an extension of the foot. A reduced shell is covered by the mantle, and the gills are located on the right side of the mantle between the shell and the right parapodia. The color of the spotted sea hare is generally a pale yellow to green color though this varies greatly with the food that they consume.

### 3.3. Preparation of Different Marine Organism Extracts

Freshly collected samples were cut into small pieces and freeze dried. The dried samples (1 kg) were macerated with ethanol for 24–48 h. After maceration, the solution was filtered and evaporated to dryness on a rotatory vacuum evaporator (Strike 202, Germany) at 40°C maximum. This constituted the crude extract (15 g) that can be stored at -20°C until used. The extract was dissolved in DMSO (10 mg/mL) and further diluted in medium to get the final testing concentrations.

### 3.4. Anticancer Screening

#### 3.4.1. Cell Culture

**HepG2**, **HCT-116**, and **MCF-7** cancer cell lines were obtained from the American Type Culture Collection (Manassas, VA, USA, and VACSERA Co., Cairo, Egypt). Those cancer cells were cultured in DMEM and were obtained from Corning Thomas Scientific (Swedesboro, NJ, USA). The reagents SRB, DMSO, and doxorubicin were purchased from Sigma-Aldrich, St. Louis, USA, while FBS was purchased from HyClone (Pittsburgh, PA, USA) and PSA was obtained from Mediatech Inc. (Herndon, VA, USA). Cancer cells were cultured in DMEM supplemented with 5% heat-inactivated FBS and 1% PSA at 37°C in a 5% CO_2_ incubator [26].

#### 3.4.2. Cell Proliferation Assay

SRB assay has been used to evaluate the cytotoxicity of all three marine extracts against **HepG2**, **HCT-116**, and **MCF-7** cancer cells. The cells of each cancer cell line were grown on 75 cm^2^ tissue culture flasks and reached its confluence usually after 24 h in a complete growth medium (DMEM). The aliquots of 100 *μ*L of cell suspension were added to each well on a 96-well sterile tissue culture plate using a multichannel pipette. The cells were incubated for 24 h at 37°C in a humidified atmosphere of 5% CO_2_. After the formation of a complete monolayer cell sheet in each well of the plate, the medium was aspirated and replaced with DMEM with 5% fetal bovine serum. Later, ***Pg*E**, ***Ha*E**, and ***Ad*E** each at various concentrations (6.25, 12.5, 25, 50, and 100 *μ*g/mL) were dispensed into a 96-tissue culture plate at 50 *μ*L/well. Another set of wells was kept including the wells of cells **Ctrl** in which 50 *μ*L of DMEM with 5% FBS is added instead of the extracts as negative **Ctrl**. The treated and untreated cells were covered with a plate sealer and allowed to grow and proliferate by further incubation of the covered plate for 24 h at 37°C in a humidified atmosphere of 5% CO_2_. Next, the medium was aspirated in order to fix cells inside the wells with 10% TCA 150 mL/well for 1 h at 4°C to reduce the SRB protein binding, and then, the wells were washed 3 times by water and stained by SRB 70 mL/well for 10 min at room temperature with 0.4% 70 mL/well to be kept in the dark. Subsequently, the wells containing cancer cells were washed with acetic acid 1% to remove the extra dye. The plates were kept in a clean place for 24 h for air drying. Afterward, a 10 mM tris base (pH 7.4) was added (50 *μ*L/well) on the shaker at 1600 rpm for 5 min. Then, the OD of each well was calculated at 570 nm with an ELISA microplate reader (EXL800, USA). The percentage of cell viability was assessed as (A570 of treated samples/A570 of untreated sample) × 100. In addition, the IC_50_ values were figured out by Microsoft Excel [2].

#### 3.4.3. Protein Extraction and Western Blotting

The media were discarded 24 h after **HepG2**, **HCT-116**, and **MCF-7** cell treatment with each *Pg*, *Ha*, and *Ad* extracts. Then, the cells were washed with cold PBS (10 mmol/L, pH 7.4) followed by incubation on an ice-cold lysis buffer (50 mmol/L Tris–HCl, 150 mmol/L NaCl, 1 mmol/L EGTA, 1 mmol/L EDTA, 20 mmol/L NaF, 100 mmol/L Na_3_VO_4_, 0.5% NP40, 1% Triton X-100, 1 mmol/L PMSF (pH 7.4)) with a freshly added inhibitor cocktail (Inhibitor Cocktail Set III, Calbiochem, La Jolla, CA) over ice for 30 minutes [27]. The cells were scraped, and the suspension was passed many times through a 21.5-gauge needle up and down in a microfuge tube to split-up any cell aggregates [28]. Then, centrifugation for lysate at 14,000 × g was performed for 25 min at 4°C, and the total cell lysate (supernatant) was stored at 80°C for further analysis [27].

**CDK2**, **PARP**, and **HRP** antimouse and anti-rabbit secondary antibodies were all obtained from Cell Signaling Technology (Beverly, MA, USA), whereas GAPDH was purchased from Santa Cruz Biotechnology, Inc. (Santa Cruz Co., Santa Cruz, CA, USA). Mini-protean precast Tris-Glycine gels were from Bio-Rad (Hercules, CA, USA). ECL detection system was from GE Healthcare (Buckinghamshire, UK). A 2% (*w*/*v*) Aqueous Solution of Gentian Violet was from Ricca Chemical Company (Arlington, TX, USA). Invitrogen Novex precast Tris-Glycine gels were from Corning. 25–40 *μ*g of protein were resolved over 8%–12% polyacrylamide gels and transferred to a nitrocellulose membrane. The blot was blocked in blocking buffer (7% nonfat dry milk/1% Tween 20; in 20 mmol/L TBS (pH 7.6)) for 1 h at room temperature, incubated with the appropriate monoclonal or polyclonal primary antibody in blocking buffer for 2 h at room temperature or overnight at 4°C, followed by incubation with an appropriate secondary antibody **HRP** conjugate. The blots were exposed to enhanced chemiluminescence (Thermo Scientific Pierce, Rockford, IL) and subjected to autoradiography using a Bio-Rad (Hercules, CA) imaging system [26, 28]. The digitalized scientific software program Quantity One (Bio-Rad) was used for the analysis of the Western blot to assess the densitometric measurements of the bands, and treatment protocol was carried out at least three times as well as each protein expression was analyzed three times with analogous results [27, 28].

#### 3.4.4. Cell Cycle Analysis

**HepG2** cancer cells were added to each well on a 6-well sterile tissue culture plate (1 × 10^5^ cells per well) and incubated in a humidified atmosphere of 5% CO_2_ for 48 h at 37°C. The cells were treated with ***Pg*E**, ***Ha*E**, and ***Ad*E** for 24 h. Next, cells were harvested and fixed using ice-cold 70% ethanol for 12 h at 4°C. Then, ethanol was removed, and the cells were washed with cold PBS and incubated for 30 min at 37°C in 0.5 mL of PBS containing 1 mg/mL RNase obtained from (Thermo Fisher Scientific Co., Waltham, MA, USA). The cells were stained with propidium iodide for 30 min in the dark. Then, the detection of DNA contents was done using a flow cytometer [2, 29].

#### 3.4.5. Determination of the Active Caspase-3

The **caspase-3** level was measured by using the Invitrogen **caspase-3** (active) Human kit from (Thermo Fisher Scientific Co., Waltham, MA, USA). Briefly, after washing the cells with PBS, they are collected and lysed by adding them to the extraction buffer containing 1 mM PMSF (stock is 0.3 M in DMSO) and protease inhibitor cocktail (e.g., Sigma-Aldrich Cat. # P-2714 (St. Louis, USA)). Later, add 500 *μ*L per 5 mL of cell extraction buffer-protease inhibitors (1 mL per 1 × 107 cells). Next, the lysate was diluted immediately prior to the assay. In the end, the OD of each well was determined within 30 minutes using a microplate reader set at 450 nm.

#### 3.4.6. Determination of Bax and Bcl-2

**HepG2** cancer cells were grown in DMEM containing 5% FBS at 37°C, and after treatment with the present marine extracts, **HepG2** suspension was tested for **Bax** and **Bcl-2** using cell extraction buffer to be lysed. This lysate was diluted in the standard diluent buffer over the range of the assay and measured for human active **Bax** and **Bcl-2** content using **Bax** ELISA (EIA-4487) kit from DRG Instruments, Ma, Germany, and Zymed **Bcl-2** ELISA Kit from Thermo Fisher Scientific Co., Waltham, MA, USA.

#### 3.4.7. Annexin-V Assay

**HepG2** cancer cells were added to each well on a 6-well sterile tissue culture plate at a concentration of 1 × 10^5^ cells per well and incubated for 48 h at 37°C in a humidified atmosphere of 5% CO_2_. The cells were treated with ***Pg*E**, ***Ha*E**, and ***Ad*E** for 24 h. Next, the cells were harvested, washed by PBS, and stained in the dark with annexin V-FITC and PI from Abcam MA, United States, in binding buffer (10 mM HEPES, 140 mM NaCl, and 2.5 mM CaCl_2_ at pH 7.4) for 15 min at room temperature and later analyzed by the flow cytometer [2, 30].

## 4. Discussion

Natural products, especially those derived from the marine environment, are recently and globally well known in the last two decades for their pharmacodynamic potential in a variety of disease therapies such as cancer. Despite the nutritional and economical importance of numerous marine organisms, the information about their bioactive compounds and biological activity is rare. Otherwise, sea cucumbers are one of the marine organisms belonging to the phylum Echinodermata; their bioactive compounds are attractive candidates for cancer chemoprevention and therapy due to previous studies that have shown the antioxidant and anticancer activities of sea cucumber compounds.

Here, two novel sea cucumber extracts ***Pg*E** and ***Ha*E** are introduced to investigate their anticancer effects as ***Sj*SG** [31], **TBL12** [32], ***HP*E** [33], ***Aj*Es** [34], **PE** [35, 36], ***Am*C**, and ***Aa*C** [37] each isolated from different sea cucumber species. In addition, **HepG2**, **HCT-116**, and **MCF-7** cancer cell lines each were exposed to ***Pg*E** and ***Ha*E** individual treatments such as **S180** [35–37], **SK-MEL-2** [34], and hepatoma 22 cells [35, 36] that each has been subjected to prior bioactive compounds of different previously investigated sea cucumbers.

Each of ***Pg*E** and ***Ha*E** was found to *in vitro* inhibit cell proliferation and induce apoptosis in **HepG2**, **HCT-116**, and **MCF-7** cancer cells, while ***Sj*SG***in vitro* inhibited angiogenesis and osteoclastogenesis and increased cytotoxicity [31]; ***Am*C** and ***Aa*C** also *in vitro* inhibited cell proliferation and induced apoptosis in **S180** cancer cells [32], ***HP*E***in vitro* induced apoptosis on **CLLB**s [33], SK-MEL-2 *in vitro* inhibited **SK-MEL-2** proliferation and metastasis [34], and **PE***in vitro* inhibited **S180** and hepatoma proliferation and increased apoptosis and antiangiogenic activity [25, 26]. However, present *in vitro* anticancer effects of ***Pg*E** or ***Ha*E** were supported by the downregulation of **CDK2**, upregulation of **PARP** in **HepG2**, **HCT-116**, and **MCF-7** cancer cells, and **Bcl-2** downturn, upregulation of **Bax** and **caspase-3**, induction of G_0_/G_1_ cell cycle arrest, and induction of apoptosis at pre**G**_**1**_ phase in **HepG2** cancer cell line, while other previously investigated sea cucumber anticancer effects were supposed to be as a result of **Bcl-2**, **STAT3**, and **MMP-9** downregulation [34]. Although quantitative real-time PCR analysis presented that the *in vivo* administration of ***Am*C** and ***Aa*C** downregulated the expression of **Bcl-2** and **Bcl-xL** while upregulated **Bax**, **Cyt C**, **caspase-3**, and **caspase-9** of the **S180** ascites tumor cells [37], the present ***Pg*E** and ***Ha*E** each *in vitro* downregulated the expression of **Bcl-2** while on the other hand upregulated **Bax** and **caspase-3** in **HepG2** cancer cell line, whereas there is another *in vivo* study that stated the enhanced antiangiogenic activity associated with inhibition of **VEGFR2** signaling of **PE** on **S180** tumor cancer cells [35, 36]. Furthermore, the clinical research about sea cucumbers' anticancer efficacy is very limited; there is only one study available to be discussed here as a total of 20 patients with high-risk **ASxMM** were given **TBL12** while **TBL12** is well tolerated, and 9 (45%) patients remain on the treatment with one MR noted [32].

Over and above that, the present study is also introducing the anticancer activity of another novel marine extract ***Ad*E** against **HepG2**, **HCT-116**, and **MCF-7** cancer cells. ***Ad*E** dose-dependently inhibited the growth of **HepG2**, **HCT-116**, and **MCF-7** cells within IC_50_ values 6.51, 5.33, and 6.87 *μ*g/mL, respectively, while the IC_50_ values for the antiproliferative activity of ***Pg*E** were 16.22, 13.34, and 18.09 *μ*g/mL, respectively, and the IC_50_ values for the antiproliferative activity of ***Ha*E** were 12.48, 10.45, and 10.36 *μ*g/mL, respectively. Therefore, those IC_50_ values of the three current extracts that ranged from 5.33 to18.09 *μ*g/mL indicated their cancer curative effects. According to the present IC_50_ values, ***Ad*E** was the highest in its cytotoxicity among all three tested extracts on **HepG2**, **HCT-116**, and **MCF-7** cells, whereas it showed a broad-spectrum activity less than 10 *μ*g/mL against the cancer cell lines. Also, ***Ad*E** was more selective towards **HCT-116** cancer cells than **HepG2** and **MCF-7** cancer cell lines. Moreover, the obtained data demonstrated that ***Ad*E** upregulated the level of the proapoptotic protein **Bax** in **HepG2** cancer cells by 9.8 folds compared to the **Ctrl** while it downregulated the level of the antiapoptotic protein **Bcl-2** by 2.7 folds compared to the **Ctrl** in the same cell line. However, ***Pg*E** upregulated the level of the proapoptotic protein **Bax** by 8.4 folds compared to the **Ctrl** while it downregulated the level of the antiapoptotic protein **Bcl-2** by 2 folds compared to the **Ctrl** in **HepG2** cancer cells, and ***Ha*E** increased the level of protein **Bax** by 5.3 folds compared to the **Ctrl** while it decreased the level of **Bcl-2** by 1.5 folds compared to the **Ctrl**. Therefore, those data indicated that ***Ad*E** is the best extract here to induce apoptosis and inhibit the proliferation of the present cancer cell lines than ***Pg*E** and ***Ha*E**. Similarly, ***Ad*E** downregulated **CDK2**; upregulated **PARP** in **HepG2**, **HCT-116**, and **MCF-7** cancer cells; upregulated **caspase-3**; inducted G_0_/G_1_ cell cycle arrest; and inducted apoptosis at the pre-**G**_**1**_ phase in **HepG2** cancer cell line.

Overall, the data mentioned above is supported by our hypothesis of ***Pg*E**, ***Ha*E**, and ***Ad*E** all have anticancer activities and hence may be promising in the future anticancer drug development for the treatment of liver, colorectal, or breast cancer.

## 5. Conclusion

The present study showed a good cancer activity of *Pearsonothuria graeffei*, *Holothuria atra*, and *Aplysia dactylomela* extracts against the three cancer cell lines, human hepatocellular carcinoma cell line (HepG2), colon carcinoma cell line (HCT-116), and mammary gland breast cancer cell line (MCF-7). And the extracts have arrest cancer cell in the G1 and S phase and caused apoptosis for cancer cells.

## Figures and Tables

**Table 1:** Cell viability of ***Pg*E**, ***Ha*E**, and ***Ad*E** on **HePG2**, **HCT-116**, and **MCF-7** cell lines.

	Cell line viability (%)	Concentration (*μ*g/mL)					
		6.25	12.5	25	50	100	IC_50_
***Pg*E**	HePG2	70.19	62.36	41.91	26.81	18.34	16.22
	HCT-116	72.37	55.29	37.66	26.45	15.25	13.34
	MCF-7	74.42	64.46	45.37	31.51	20.48	18.09
***Ha*E**	HePG2	65.81	50.74	39.94	25.64	14.37	12.48
	HCT-116	63.44	48.38	38.74	23.29	12.37	10.45
	MCF-7	66.61	51.84	40.36	30.19	12.55	10.36
***Ad*E**	HePG2	50.11	39.47	27.87	16.62	8.58	6.51
	HCT-116	49.78	37.26	25.69	14.27	6.16	5.33
	MCF-7	50.53	40.09	30.36	18.78	8.33	6.87

## Data Availability

Data are available from the corresponding author upon reasonable request.

## Abbreviations

*Aa*C: *Asterias amurensis* cerebrosides
*Ad*: *Aplysia dactylomela*
*Ad*E: *Aplysia dactylomela* crude extract
*Aj*Es: *Apostichopus japonicus* extracts
*Am*C: *Acaudina molpadioides* cerebrosides
ASx: Asymptomatic
Cat.: Catalog number
CLLB: Chronic lymphocytic leukemia B-lymphocytes
*Ha*: *Holothuria atra*
*Ha*E: *Holothuria atra* crude extract
HCT-116: Colon carcinoma cell line
*HP*E: *Holothuria parva* methanolic extract
HepG2: Human hepatocellular carcinoma cell line
HRP: Horseradish peroxidase-conjugated
MCF-7: Mammary gland breast cancer cell line
MM: Multiple myeloma
MMP-9: Matrix metalloproteinase-9
MR: Minimal response
PARP: Poly ADP ribose polymerase
PE: Philinopside E
*PG*: *Pearsonothuria graeffei*
*Pg*E: *Pearsonothuria graeffei E*
PMSF: Phenylmethylsulfonyl fluoride
PSA: Penicillin-streptomycin-amphotericin B
SRB: Sulphorhodamine-B
STAT3: Signal transducer and activator of transcription 3
*Sj*SG: Sphingolipids/glycosides of sea cucumber *Stichopus japonicus*
SK-MEL-2: Skin melanoma cancer cell line-2
S180: Murine sarcoma cancer cell line-180
TBL12: Drug extract of sea cucumber
VEGFR2: Vascular endothelial growth factor-2

## Supplementary Materials

Supplementary Materials Attached is the GC Mass as supplementary data for this research work.
