# Supplementary material for: Antiproliferative Activity, Proapoptotic Effect, and Cell Cycle Arrest in Human Cancer Cells of Some Marine Natural Product Extract
Source: Oxid Med Cell Longev. 2020 Nov 25;2020:7948705. doi: 10.1155/2020/7948705 (PMC7714591; doi:10.1155/2020/7948705)
Supplement: Supplementary Materials — Attached is the GC Mass as supplementary data for this research work. [file 7948705.f1.pdf]

Compound	Formula	RT (min)	MF	RMF	Prob (%)	CAS #	Lib	ID	MW (g/M)	Ext	Structure
**8-Azabicyclo[3.2.1]octan -3-ol, 6-methoxy-8-methyl-**	$C_9H_{17}NO_2$	13.351	596	817	7.27%	54725-47-2	ML	81303	171	*Ad*E	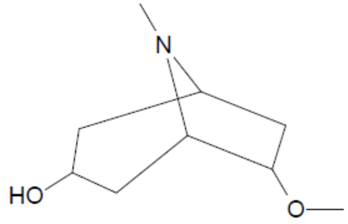
**d-Mannose**	$C_6H_{12}O_6$	15.298	705	777	22.3%	3458-28-4	ML	37454	180	*Ad*E	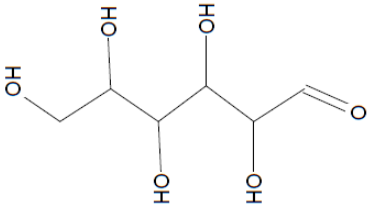
**2-Tridecanone**	$C_{13}H_{26}O$	20.455	746	827	5.68%	593-08-8	RL	6452	198	*Ha*E	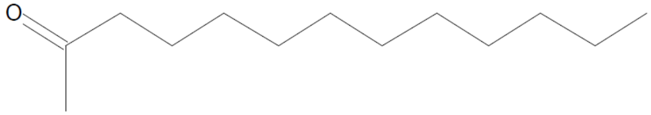
**Cyclohexane, 1-ethenyl-1-methyl-2-(1-methylethenyl)-4-(1-methylethylidene)-**	$C_{15}H_{24}$	12.670	736	891	7.65%	3242-08-8	RL	17769	204	*Ad*E	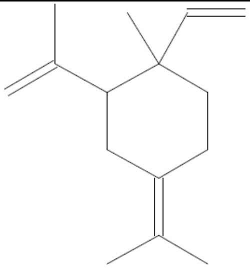

**γ-Elemen**	$C_{15}H_{24}$	12.670	733, 717 & 748	882, 944 & 911	11.5%	29873-99-2	ML & RL	91836, 1313 & 17755	204	*Ad*E	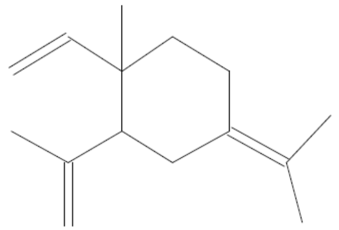
**4,5-di-epi-aristolochene**	$C_{15}H_{24}$	12.670	710	828	2.94%	—	ML	70251	204	*Ad*E	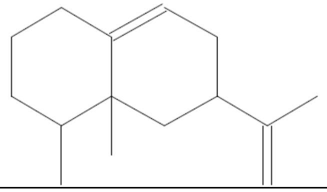
**6-(3-Isopropenylcycloprop-1-enyl)-6-methylhept-3-en-2-one**	$C_{14}H_{20}O$	15.500	635	693	5.85%	—	ML	91953	204	*Ad*E	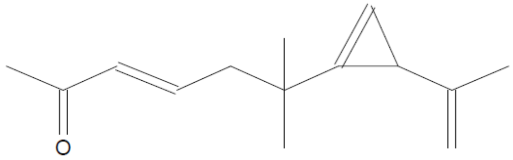
**β-Guaiene**	$C_{15}H_{24}$	15.956	701	763	6.59%	88-84-6	ML	133155	204	*Ad*E	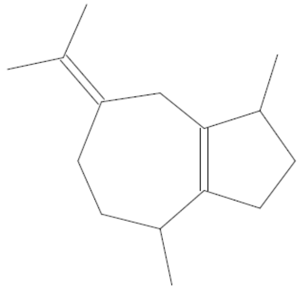
**Naphthalene, decahydro-4a-methyl-1-methylene-7-(1-methylethylidene)-, (4aR-trans)-**	$C_{15}H_{24}$	15.956	691	805	4.34%	515-17-3	RL	23149	204	*Ad*E	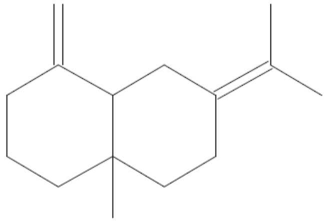

**d-Glycero-l-gluco-heptose**	$C_7H_{14}O7$	15.500 & 15.725	640 & 621	706 & 701	7.26% & 4.72%	—	ML	36984	210	*Ad*E	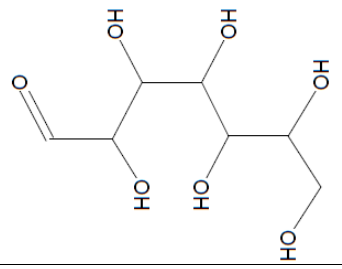
**2-Tetradecanone**	$C_{14}H_{28}O$	20.455	759 & 754	910 & 854	9.43%	2345-27-9	RL	6447& 6448	212	*Ha*E	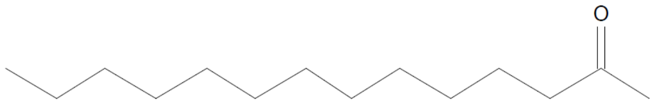
**1H-Cycloprop[e]azulen-7-ol, decahydro-1,1,7-trimethyl-4-methylene-, [1ar-(1aα,4aα,7β,7aβ,7bα)]-**	$C_{15}H_{24}O$	16.060 & 18.630	755 , 767 & 762	884, 865 & 799	16.9% & 7.32%	6750-60-3	RL & ML	1833 & 6115	220	*Ad*E	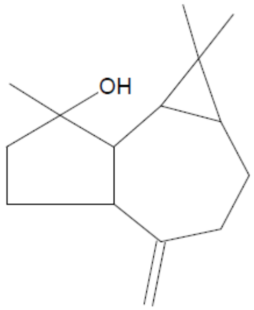
**(-)-Spathulenol**	$C_{15}H_{24}O$	16.060 , 18.630 & 18.751	730 , 776 & 720	783, 813 & 781	5.16% , 10.1% & 3.02%	77171-55-2	ML	5991			
**Tricyclo[5.2.2.0(1,6)]undecan-3-ol, 2-methylene-6,8,8-trimethyl-**	$C_{15}H_{24}O$	16.060 & 18.630	722 & 761	806 & 817	3.68% & 5.67%	—	ML	3909	220	*Ad*E	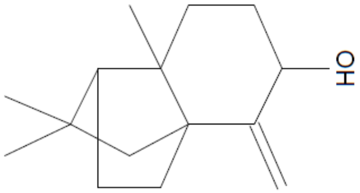

**Ledene oxide-(II)**	$C_{15}H_{24}O$	16.060, 18.630 & 18.751	720, 762 & 718	769, 796 & 776	3.39%, 5.90% & 2.78%	—	ML	5399	220	*Ad*E	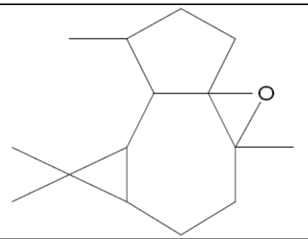
**Isoaromadendrene epoxide**	C15H24O	18.630 & 18.751	780 & 737	841 & 838	11.9% & 5.80%	—	ML	2203	220	*Ad*E	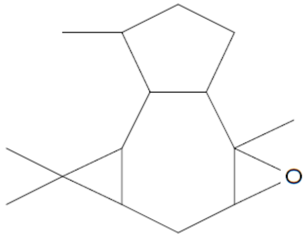
**α-acorenol**	C15H26O	12.670 & 15.956	718 & 714	819 & 785	3.94% & 10.2%	—	ML	89275	222	*Ad*E	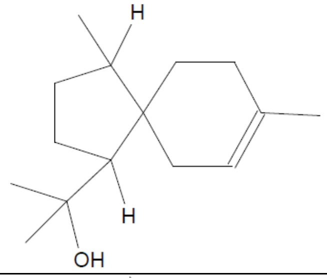
**4aH-Cycloprop[e]azulen-4a-ol, decahydro-1,1,4,7-tetramethyl-, [1aR-(1aα,4β,4aβ,7α,7aβ,7bα)]-**	$C_{15}H_{26}O$	15.130	716 & 680	825 & 779	24.7%	5986-49-2	RL	16287	222	*Ad*E	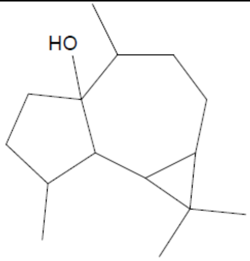

**4aH-cycloprop[e]azulen-4a-ol, decahydro-1,1,4,7-tetramethyl-**	$C_{15}H_{26}O$	15.130	684	780	6.60%	95975-84-1	ML	79162	222	*Ad*E	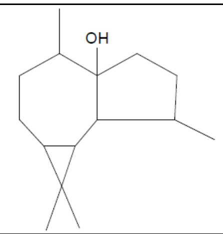
**1H-Cycloprop[e]azulen-4-ol, decahydro-1,1,4,7-tetramethyl-, [1aR-(1aα,4β,4aβ,7α,7aβ,7bα)]-**	$C_{15}H_{26}O$	15.130	677	839	4.90%	552-02-3	ML	5852	222	*Ad*E	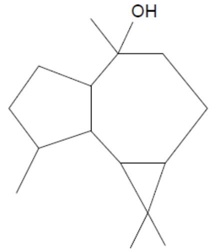
**7-epi-cis-sesquisabinene hydrate**	$C_{15}H_{26}O$	15.130	677	757	4.90%	—	ML	33030	222	*Ad*E	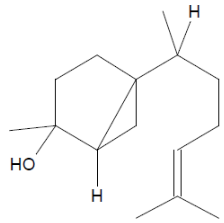
**4-epi-cubedol**	$C_{15}H_{26}O$	15.956	697	762	5.57%	—	ML	133124	222	*Ad*E	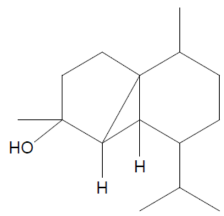
**cubedol**	$C_{15}H_{26}O$	15.956	693	772	4.70%	—	ML	133141			

**1,4-Methanoazulen-3-ol, decahydro-1,5,5,8a-tetramethyl-, [1S-(1α,3β,3aβ,4α,8aβ)]-**	C15H26O	17.336	656	693	2.77%	99481-30-8	ML	50278	222	*Ad*E	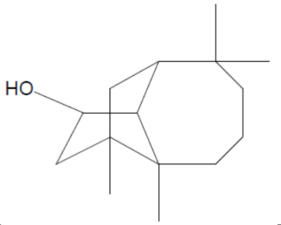
**2-Pentadecanone**	$C_{15}H_{30}O$	20.455	752	834	7.22%	2345-28-0	ML	25959	226	*Ha*E	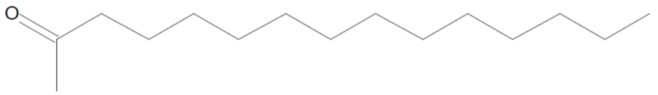
**Cedran-diol, 8S,13-**	$C_{15}H_{26}O_2$	16.060 & 18.751	719 & 728	779 & 791	3.26% & 4.16%	88588-48-1	ML	5710	238	*Ad*E	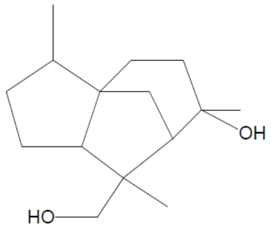
**Methyl tetradecanoate**	$C_{15}H_{30}O_2$	20.894	748	856	10.7%	124-10-7	RL	9796	242	*Pg*E	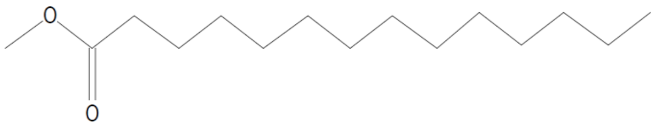
**2-Hexadecanol**	$C_{16}H_{34}O$	23.805	721	786	4.86%	14852-31-4	RL	3801	242	*Pg*E	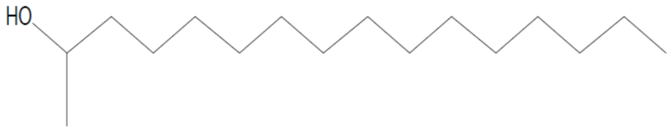

**Ethyl tridecanoate**	$C_{15}H_{30}O_2$	22.095	794 & 791	863 & 849	15.2%	28267-29-0	RL& ML	11989& 52397	242	*Ha*E	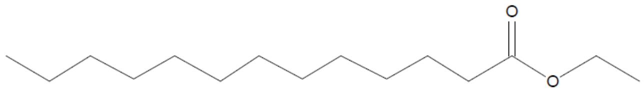
**Methyl 6-O-[1-methylpropyl]-β-d-galactopyranoside**	$C_{11}H_{22}O_6$	15.298	663	760	4.05%	—	ML	23313	250	*Ad*E	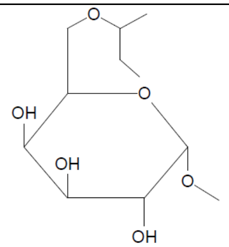
**Tertbutyloxyformamide, N-methyl-N-[4-(1-pyrrolidinyl)-2-butynyl]-**	$C_{14}H_{24}N_2O_2$	13.351	614	652	14.2%	124045-68-7	ML	34145	252	*Ad*E	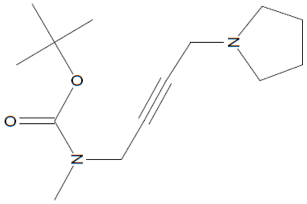
**Spiro[tricyclo[4.4.0.0(5,9)]decane-10,2'-oxirane], 1-methyl-4-isopropyl-7,8-dihydroxy-, (8S)-**	$C_{15}H_{24}O_3$	15.500	644	675	8.60%	—	ML	142870	252	*Ad*E	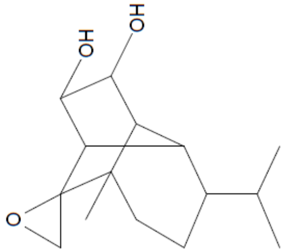

**5,6,7,8,9,10-Hexahydro-9-methyl-spiro[2H-1,3-benzoxazine-4,1'-cyclohexane]-2-thione**	$C_{14}H_{23}NOS$	13.351	599	639	8.23%	—	ML	131040	253	*Ad*E	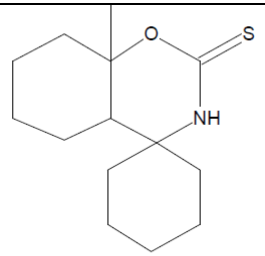
**2-Heptadecanone**	$C_{17}H_{34}O$	20.455	791	825	31.1%	2922-51-2	RL	2300	254	*Ha*E	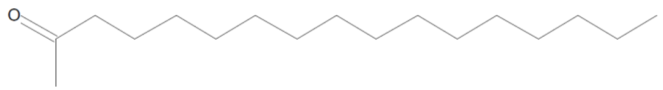
**Tetradecanoic acid, 12-methyl-, methyl ester**	$C_{16}H_{32}O_2$	20.894 & 24.607	743 & 705	774 & 759	8.64% & 5.32%	5129-66-8	ML	40491	256	*Pg*E	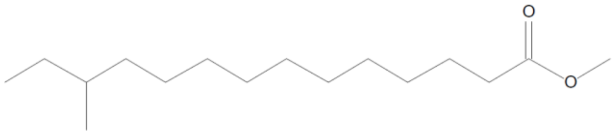
**1-Hexadecanol, 2-methyl-**	$C_{17}H_{36}O$	23.805 & 29.303	722 & 694	776 & 735	5.06% & 5.66%	2490-48-4	ML	22458	256	*Pg*E & *Ha*E	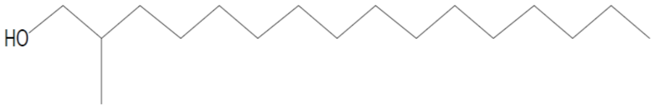
**tert-Hexadecanethiol**	$C_{16}H_{34}S$	29.303	711	761	10.7%	25360-09-2	ML	23376	258	*Ha*E	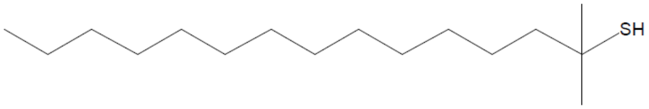
**14-Octadecenal**	$C_{18}H_{34}O$	22.673	803	826	5.96%	56554-89-3	ML	46618	266	*Pg*E	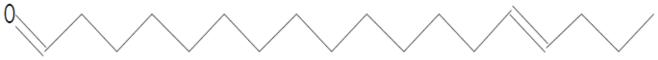

**16-Octadecenal**	$C_{18}H_{34}O$	22.673	802	825	5.73%	56554-87-1	ML	46617	266	*Pg*E	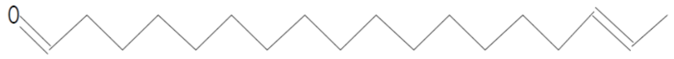
**10-Octadecenal**	$C_{18}H_{34}O$	22.673	795	819	4.39%	56554-92-8	ML	22746	266	*Pg*E	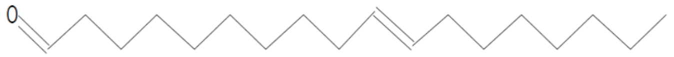
**12-Octadecenal**	$C_{18}H_{34}O$	22.673	794	822	4.22%	56554-91-7	ML	46620	266	*Pg*E	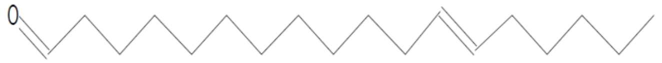
**5-Octadecenal**	$C_{18}H_{34}O$	23.805	728	779	6.53%	56554-88-2	ML	22758	266	*Pg*E	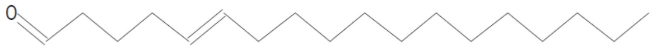
**4-Octadecenal**	$C_{18}H_{34}O$	23.805	720	771	4.67%	56554-98-4	ML	22760	266	*Pg*E	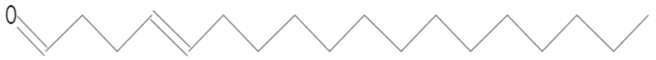
**Hexadecanoic acid, methyl ester**	$C_{17}H_{34}O_2$	20.894	751	836	12.1%	112-39-0	RL	9767	270	*Pg*E	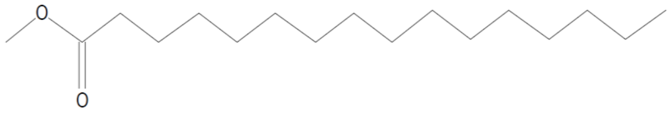
	$C_{17}H_{34}O_2$	20.894	739	794	12.1%	112-39-0	ML	40690			
**Pentadecanoic acid, 14-methyl-, methyl ester**	$C_{17}H_{34}O_2$	20.894	738	830	6.96%	5129-60-2	ML	40704	270	*Pg*E	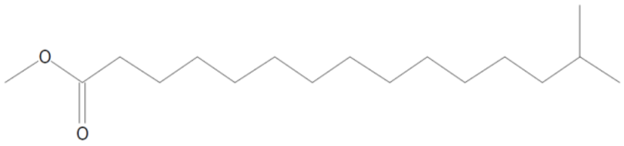

**Tridecanoic acid, 13-formyl-, ethyl ester**	$C_{16}H_{30}O_3$	25.814	659	709	2.32%	101434-22-4	ML	52481	270	*Pg*E	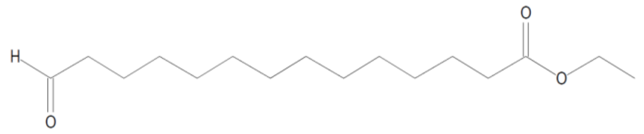
**Pentadecanoic acid, ethyl ester**	$C_{17}H_{34}O_2$	22.095	791	841	13.4%	41114-00-5	ML	52728	270	*Ha*E	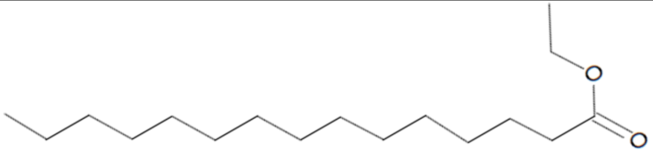
**Undecanoic acid, 11-bromo-, methyl ester**	$C_{12}H_{23}BrO_2$	17.336	666	715	4.16%	6287-90-7	RL	9817	278	*Ad*E	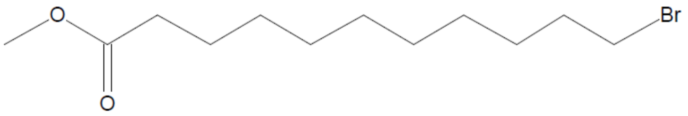
**Desulphosinigrin**	$C_{10}H_{17}NO_6S$	15.298, 15.500 & 15.725	704, 660 & 669	776, 730 & 760	21.5%, 14.9% & 27.4%	5115-81-1	ML	28432	279	*Ad*E	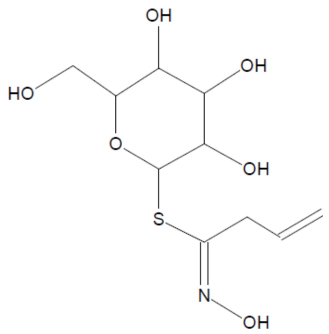
**9,12-Octadecadienoic acid (Z,Z)-**	$C_{18}H_{32}O_2$	37.203	709	751	3.03%	60-33-3	RL	7667	280	*Ha*E	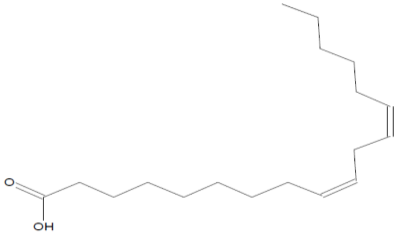

**Ethyl 9,12-hexadecadienoate**	C18H32O2	37.203	708	793	2.92%	—	ML	30104	280	*Ha*E	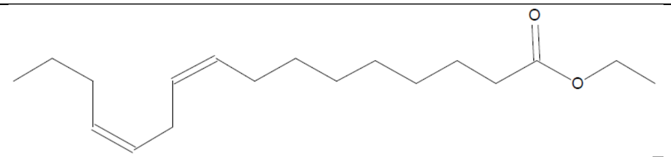
**2-Nonadecanone**	$C_{19}H_{38}O$	20.455	771	906	14.2%	629-66-3	ML	25712	282	*Ha*E	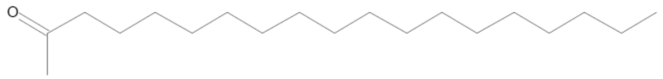
**cis-Vaccenic acid**	$C_{18}H_{34}O_2$	25.353	763	791	5.10%	506-17-2	ML	18782	282	*Ha*E	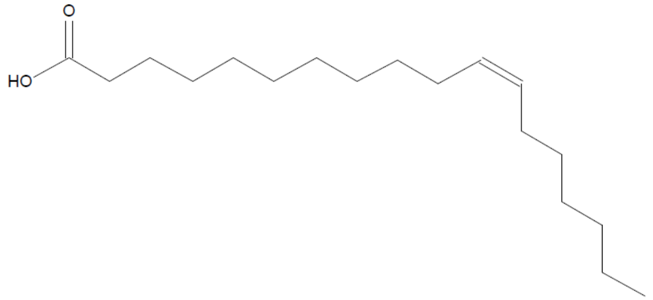
**trans-13-Octadecenoic acid**	$C_{18}H_{34}O_2$	25.353	760	792	4.53%	693-71-0	ML	18062	282	*Ha*E	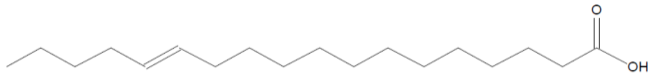
**Ethyl 9-hexadecenoate**	$C_{18}H_{34}O_2$	34.443	659	722	3.24%	54546-22-4	ML	19602	282	*Ha*E	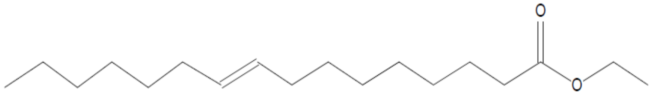
**Hexadecanoic acid, 14-methyl-, methyl ester**	$C_{18}H_{36}O_2$	20.894	738	748	6.96%	2490-49-5	ML	40703	284	*Pg*E	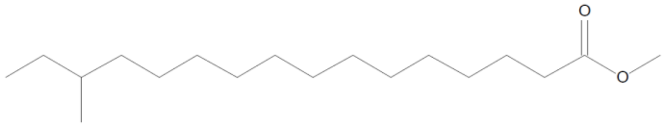
**Hexadecanoic acid, ethyl ester**	$C_{18}H_{36}O_2$	22.101	814	827	56.5%	628-97-7	RL	12003	284	*Pg*E	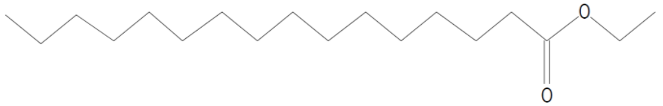

			733	790	56.5%	628-97-7	ML	52733			
			724	830	56.5%	628-97-7	RL	12002			
**Hexadecanoic acid, 14-methyl-, methyl ester**	$C_{18}H_{36}O_2$	24.607	710	735	6.55%	2490-49-5	ML	40703			
**Hexadecanoic acid, ethyl ester**	$C_{18}H_{36}O_2$	25.814	761	818	60.1%	628-97-7	RL	12003			
		22.095	797	812	17.2%	628-97-7	ML	52733	284	*Ha*E	
		22.095	789	789	17.2%	628-97-7	RL	12003			
**Methyl 16-hydroxy-hexadecanoate**	$C_{17}H_{34}O_3$	24.607	709	814	6.30%	21987-14-4	ML	19119	286	*Pg*E	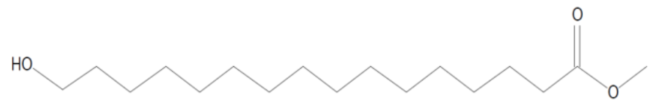
**10,13-Octadecadiynoic acid, methyl ester**	$C_{19}H_{30}O_2$	16.060	726	745	4.36%	18202-24-9	ML	55439	290	*Ad*E	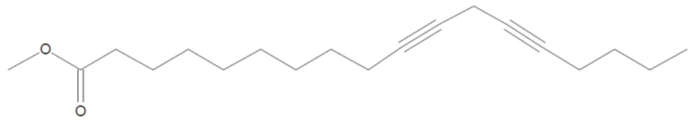

**6,9,12-Octadecatrienoic acid, methyl ester**	$C_{19}H_{32}O_2$	27.125	680	713	4.21%	2676-41-7	ML	30024	292	*Pg*E	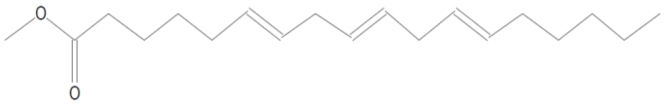
**Methyl 6,11-octadecadienoate**	$C_{19}H_{34}O_2$	37.203	708	785	2.92%	—	ML	45926	294	*Ha*E	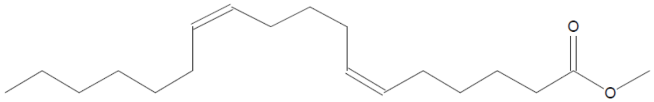
**Cyclopentanetridecanoic acid, methyl ester**	$C_{19}H_{36}O_2$	24.607	702	840	4.70%	24828-61-3	ML	40685	296	*Pg*E	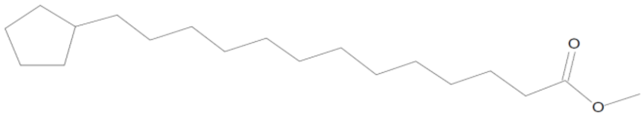
**16-Octadecenoic acid, methyl ester**	$C_{19}H_{36}O_2$	24.821	675	699	3.55%	56554-49-5	ML	18104	296	*Pg*E	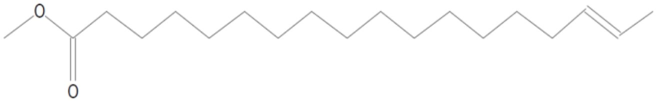
**10-Octadecenoic acid, methyl ester**	$C_{19}H_{36}O_2$	25.353 & 17.336	762 & 659	775 & 668	4.90% & 3.14%	13481-95-3	ML	18073	296	*Ha*E & AdE	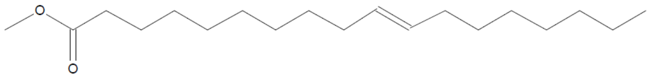
**11-Octadecenoic acid, methyl ester**	C19H36O2	25.353	761	785	4.71%	52380-33-3	ML	17844	296	*Ha*E	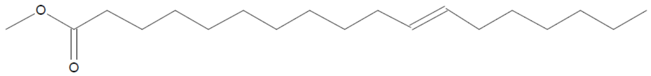
**Heptadecanoic acid, ethyl ester**	$C_{19}H_{38}O_2$	22.101	727	804	4.98%	14010-23-2	ML	52724	298	*Pg*E	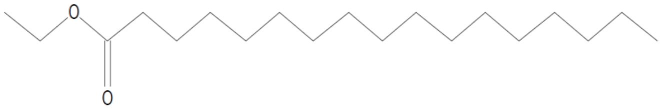
**Ethyl 14-methyl-hexadecanoate**	$C_{19}H_{38}O_2$	22.101 & 25.814	725 & 697	785 & 790	4.59% & 9.87%	2490-49-5	ML	52472	298	*Pg*E	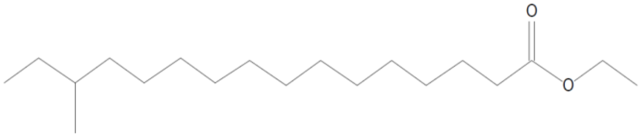

**Heptadecanoic acid, ethyl ester**	$C_{19}H_{38}O_2$	25.814	654	781	1.87%	14010-23-2	ML	52724	298	*Pg*E	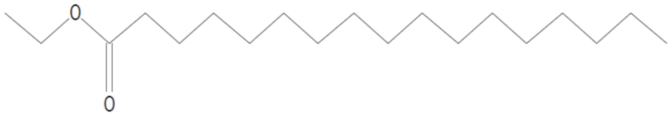
**Oxiraneoctanoic acid, 3-octyl-, cis-**	$C_{18}H_{34}O_3$	20.351	724	743	21.2%	24560-98-3	RL	4389	298	*Ad*E	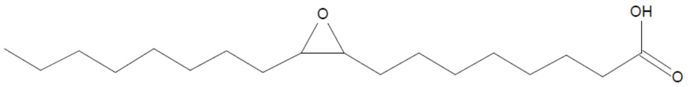
**Benz[e]azulene-3,8-dione, 3a,4,6a,7,9,10,10a,10b-octahydro-3a,10a-dihydroxy-5-(hydroxymethyl)-2,10-dimethy**	$C_{17}H_{22}O_5$	53.432	650	701	5.62%	25578-89-6	ML	166443	306	*Ha*E	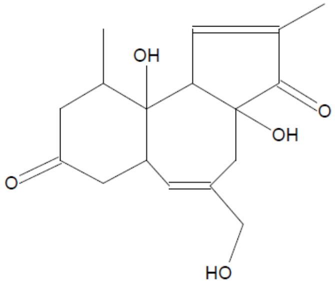
**2H-Cyclohepta[b]furan-2-one, 6-[1-(acetyloxy)-3-oxobutyl]-3,3a,4,7,8,8a-hexahydro-7-methyl-3-methylene-**	$C_{17}H_{22}O_5$	16.609	709	759	6.57%	580-49-4	RL	1908	306	*Ad*E	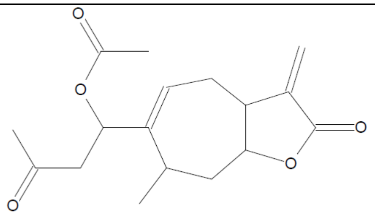
**Cyclopropaneoctanoic acid, 2-octyl-, methyl ester**	$C_{20}H_{38}O_2$	24.607 & 24.821	702 & 675	723 & 708	4.70% & 3.55%	10152-62-2	RL	4725	310	*Pg*E	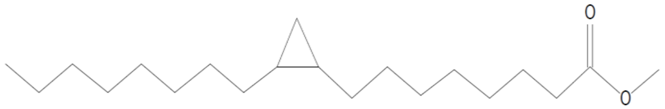

**Cyclopropanepentanoic acid, 2-undecyl-, methyl ester, trans-**	$C_{20}H_{38}O_2$	24.607	693	720	3.41%	42199-20-2	ML	2255	310	*Pg*E	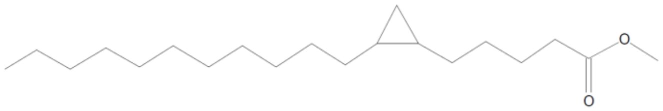
**Ethyl Oleate**	$C_{20}H_{38}O_2$	25.353 , 25.353 & 34.443	759, 756 & 652	782, 773 & 717	4.35% , 4.35% & 2.43%	111-62-6	RL	4704& 4703	310	*Ha*E	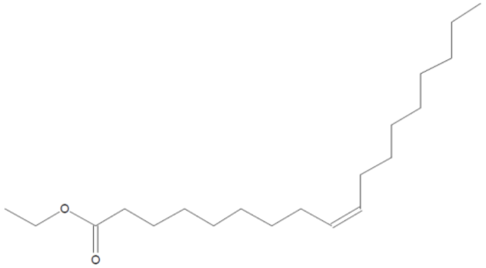
**cis-13-Eicosenoic acid**	$C_{20}H_{38}O_2$	34.443	662	736	3.67%	17735-94-3	ML	18940	310	*Ha*E	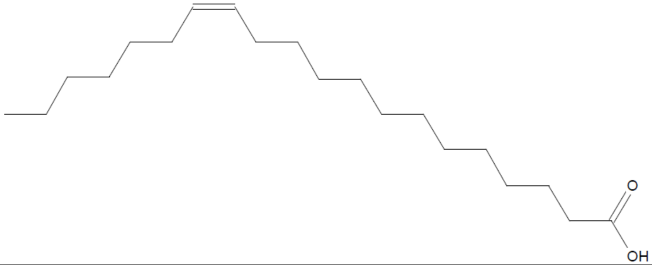
**Methyl 15-hydroxy-9,12-octadecadienoate**	$C_{19}H_{34}O_3$	37.203	711	761	3.28%	—	ML	19181	310	*Ha*E	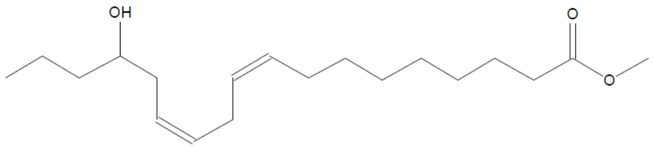
**Ethanol, 2-(9-octadecenyloxy)-, (Z)-**	$C_{20}H_{40}O_2$	22.673	825	838	15.1%	5353-25-3	ML	19248	312	*Pg*E	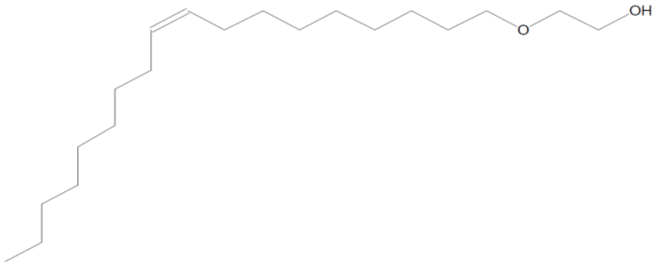

**Oxiraneundecanoic acid, 3-pentyl-, methyl ester, cis-**	$C_{19}H_{36}O_3$	24.821 & 17.336	682 & 665	705 & 674	4.71% & 4.00%	8520-30-8	ML	17859	312	*Pg*E& *Ad*E	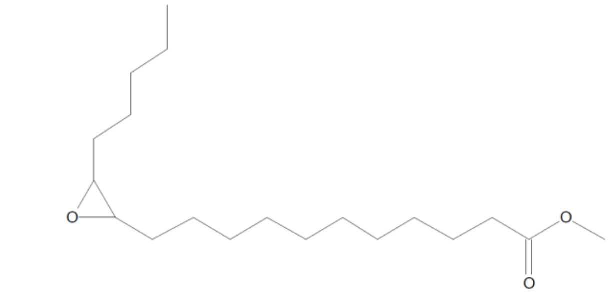
**Oxiraneundecanoic acid, 3-pentyl-, methyl ester, trans-**	$C_{19}H_{36}O_3$	24.821 & 17.336	681 & 670	704 & 679	4.52% & 4.92%	38520-31-9	ML	17744	312	*Pg*E& *Ad*E	
**Octadecanoic acid, ethyl ester**	$C_{20}H_{40}O_2$	25.814 & 22.095	660 & 778	702 & 834	2.41% & 8.68%	111-61-5	RL	12004& 12005	312	*Pg*E& *Ha*E	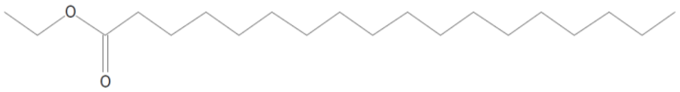
**Ethanol, 2-(octadecyloxy)-**	$C_{20}H_{42}O_2$	22.095	704	735	8.23%	2136-72-3	ML	22653	314	*Ha*E	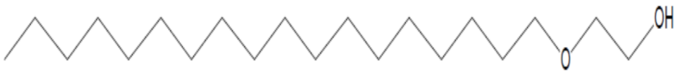
**Erythro-9,10-dihydroxyoctadecanoic acid**	C18H36O4	20.351	691	727	5.44%	3639-32-5	ML	127728	316	*Ad*E	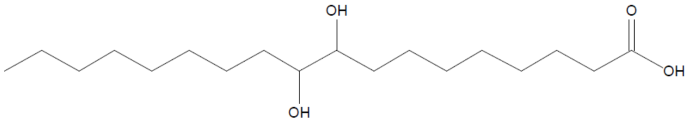

**5,8,11,14-Eicosatetraenoic acid, methyl ester, (all-Z)-**	$C_{21}H_{34}O_2$	27.125	680	805	4.21%	2566-89-4	RL	10455	318	*Pg*E	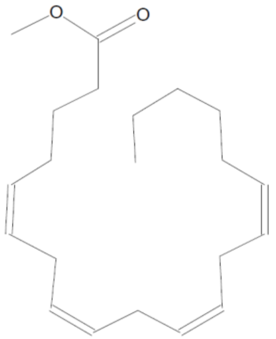
**Androstan-17-one, 3-ethyl-3-hydroxy-, (5α)-**	$C_{21}H_{34}O_2$	16.609 & 18.751	703 & 722	746	5.12% & 3.27%	57344-99-7	ML	55206	318	*Ad*E	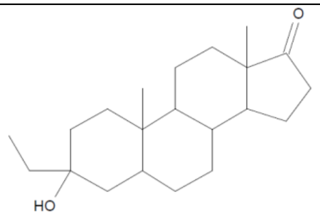
**7,10,13-Eicosatrienoic acid, methyl ester**	$C_{21}H_{36}O_2$	27.125	687	715	5.66%	30223-51-9	RL	7646	320	*Pg*E	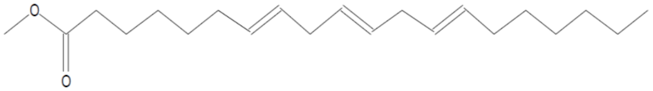
**Trinexapac-ethyl, trimethylsilyl ether**	$C_{16}H_{24}O_5Si$	42.216	593	652	9.12%	—	ML	39428	324	*Pg*E	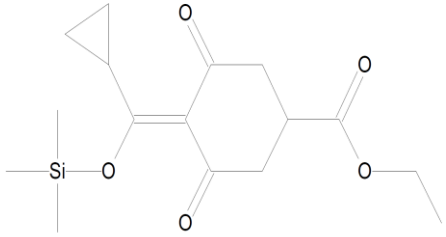
**Nonadecanoic acid, ethyl ester**	$C_{21}H_{42}O_2$	22.101 & 25.814	723 & 676	772 & 765	4.23% & 4.19%	18281-04-4	ML	52717	326	*Pg*E	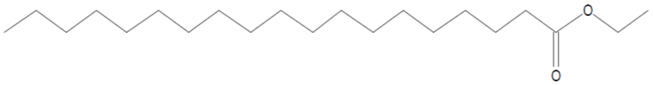

**6-Amino-5-cyano-4-(5-cyano-2,4-dimethyl-1H-pyrrol-3-yl)-2-methyl-4H-pyran-3-carboxylic acid ethyl ester**	$C_{17}H_{18}N_4O_3$	29.846, 30.354, 42.216 & 46.889	589, 584, 572 & 576	647, 642, 645 & 636	3.12%, 2.31%, 4.41% & 4.65%	—	ML	166229	326	*Pg*E	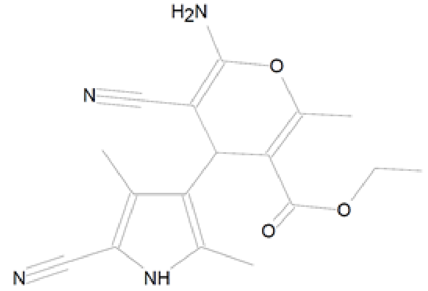
**Androst-5,7-dien-3-ol-17-one, acetate**	$C_{21}H_{28}O_3$	29.846 & 30.354	580 & 589	615 & 631	2.26% & 2.87%	—	ML	13366	328	*Pg*E	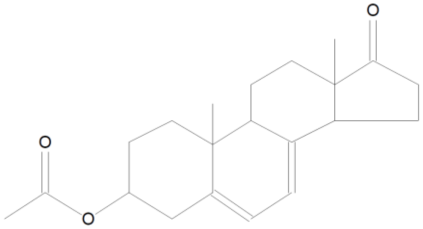
**Octadecanoic acid, 9,10-dihydroxy-, methyl ester**	$C_{19}H_{38}O_4$	20.351	680	726	3.73%	1115-01-1	RL	4577	330	*Ad*E	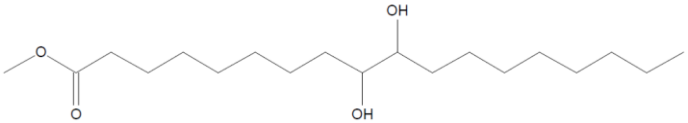
**5,8,11,14-Eicosatetraenoic acid, ethyl ester, (all-Z)-**	$C_{22}H_{36}O_2$	27.125	690	761	6.40%	1808-26-0	ML	44292	332	*Pg*E	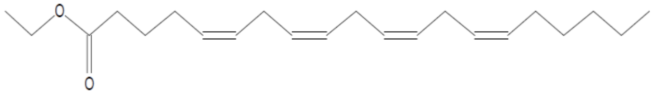
**Ethyl 5,8,11,14-eicosatetraenoate**	$C_{22}H_{36}O_2$	27.125	682	758	4.56%	95285-77-1	ML	44584	332	*Pg*E	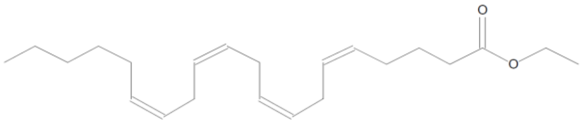

**17α-Hydroxypregnenolone**	$C_{21}H_{32}O_3$	40.316	660	692	4.74%	—	ML	9645	332	*Ha*E	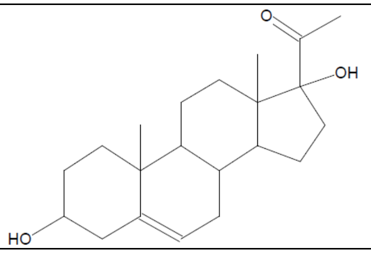
**2H-Pyran, 2-(7-heptadecynyloxy)tetrahydro-**	$C_{22}H_{40}O_2$	16.609	694	727	3.48%	56599-50-9	ML	17855	336	*Ad*E	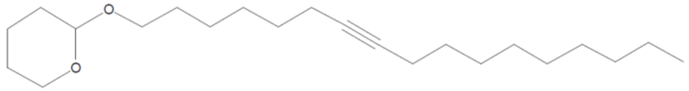
**Arsenous acid, tris(trimethylsilyl) ester**	$C_9H_{27}AsO_3Si_3$	46.889	580	840	5.50%	55429-29-3	ML	166530	342	*Pg*E	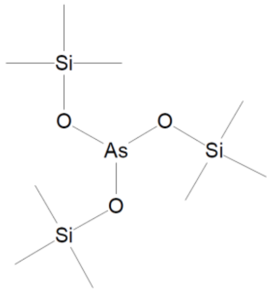
**β-D-Glucopyranose, 4-O-β-D-galactopyranosyl-**	$C_{12}H_{22}O_{11}$	15.298, 15.500 & 15.725	692, 684, 646 & 641	746, 767, 692 & 706	14.3%, 9.31% & 9.67%	5965-66-2	ML& RL	36989& 8898	342	*Ad*E	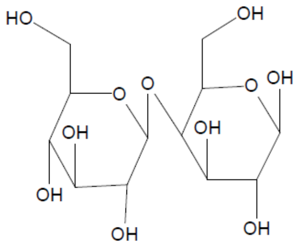

**Maltose**	$C_{12}H_{22}O_{11}$	15.298	661	733	3.73%	69-79-4	ML	37466	342	*Ad*E	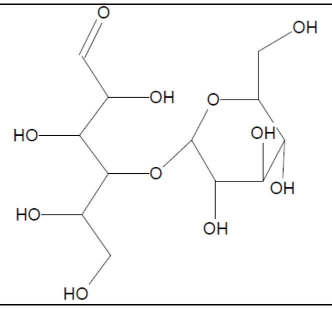
**Octadecanal, 2-bromo-**	$C_{18}H_{35}BrO$	22.673	803	811	5.96%	56599-95-2	ML	7263	346	*Pg*E	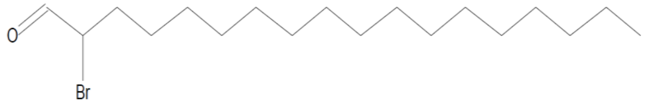
**Benz[e]azulene-3,8-dione, 5-[(acetyloxy)methyl]-3a,4,6a,7,9,10,10a,10b-octahydro-3a,10a-dihydroxy-2,10-dime**	$C_{19}H_{24}O_6$	53.432	657	706	7.56%	25536-74-7	ML	12792	348	*Ha*E	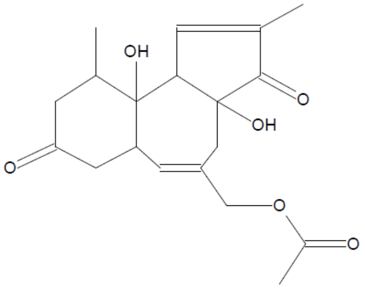
**Methyl 5,9-docosadienoate**	$C_{23}H_{42}O_2$	37.203	725	817	5.25%	—	ML	45683	350	*Ha*E	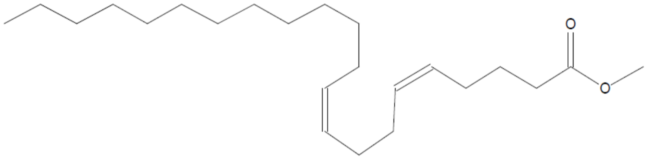

**Propanoic acid, 2-methyl-, (dodecahydro-6a-hydroxy-9a-methyl-3-methylene-2,9-dioxoazuleno[4,5-b]furan-6-yl)m**	$C_{19}H_{26}O_6$	16.609	699	733	4.32%	33649-17-1	ML	7019	350	*Ad*E	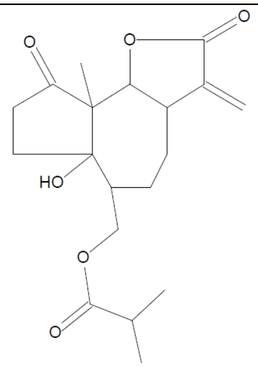
**9,10-Secochola-5,7,10(19)-trien-24-al, 3-hydroxy-, (3β,5Z,7E)-**	$C_{24}H_{36}O_2$	16.609	723	804	10.5%	40013-88-5	ML	88071	356	*Ad*E	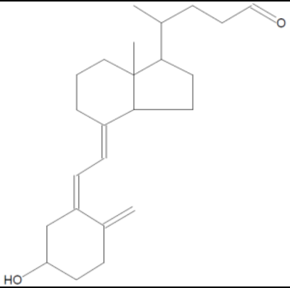
**3-[(8a-Methyl-5-methylene-2-oxo-dodecahydro-naphtho[2,3-b]furan-3-ylmethyl)-amino]-azepan-2-one**	$C_{21}H_{32}N_2O_3$	13.351	594	650	6.71%	—	ML	49312	360	*Ad*E	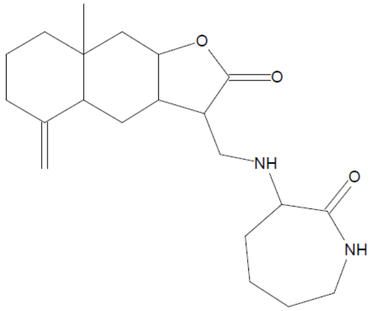
**Cyclopropanedodecanoic acid, 2-octyl-, methyl ester**	$C_{24}H_{46}O_2$	24.821	693	712	6.86%	10152-65-5	ML	2537	366	*Pg*E	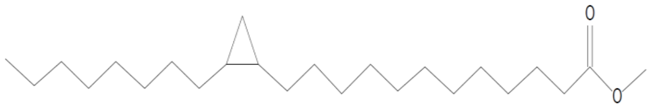

**4,6,8(14)-Cholestatriene**	$C_{27}H_{42}$	29.846	579	646	2.18%	51028-26-3	ML	13246	366	*Pg*E	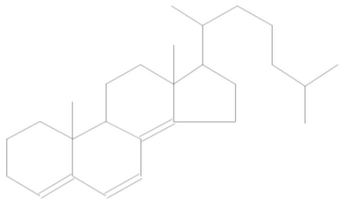
**Octadecane, 3-ethyl-5-(2-ethylbutyl)-**	$C_{26}H_{54}$	44.128	681	686	3.65%	55282-12-7	ML	7471	366	*Ha*E	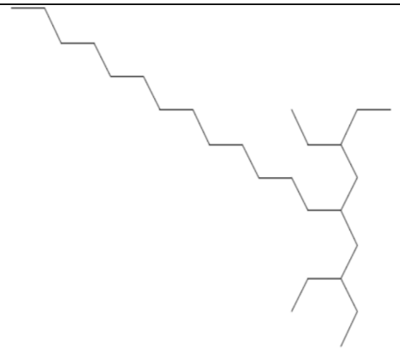
**Cyclopropanedodecanoic acid, 2-octyl-, methyl ester**	C24H46O2	17.336	675	682	6.11%	10152-65-5	ML	2537	366	*Ad*E	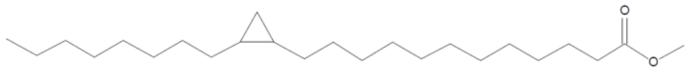
**Cyclopropanebutanoic acid, 2-[[2-[[2-[(2-pentylcyclopropyl)methyl]cyclopropyl]methyl]cyclopropyl]methyl]-, methyl ester**	$C_{25}H_{42}O_2$	24.821	706	769	10.6%	56051-53-7	ML	40568	374	*Pg*E	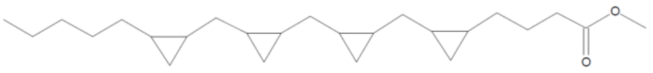
**3β-Hydroxy-5-cholen-24-oic acid**	$C_{24}H_{38}O_3$	44.128 & 47.068	664 & 671	676 & 717	3.09% & 1.39%	5255-17-4	ML	75250	374	*Ha*E	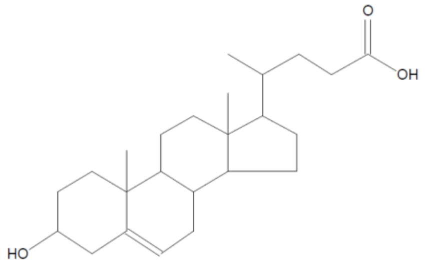

**Cyclopropanebutanoic acid, 2-[[2-[[2-[(2-pentylcyclopropyl)methyl]cyclopropyl]methyl]cyclopropyl]methyl]-, methy**	$C_{25}H_{42}O_2$	16.609	705	759	5.55%	56051-53-7	ML	40568	374	*Ad*E	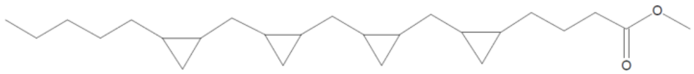
**Desoximetasone**	$C_{22}H_{29}FO_4$	15.130	682	708	6.08%	382-67-2	ML	93616	376	*Ad*E	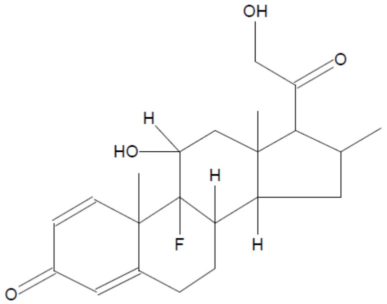
**15-Tetracosenoic acid, methyl ester, (Z)-**	$C_{25}H_{48}O_2$	34.443	666	695	4.34%	2733-88-2	RL	4753	380	*Ha*E	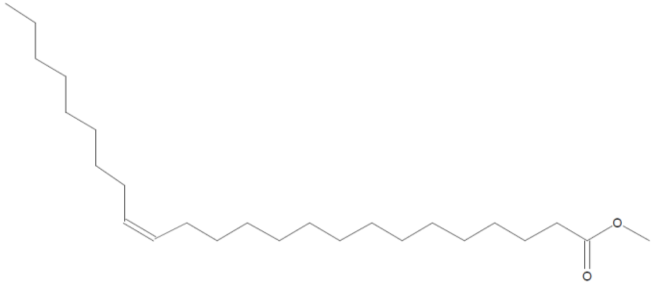
**β-D-Mannofuranoside, farnesyl-**	$C_{21}H_{36}O_6$	15.956	693	745	4.70%		ML	31505	384	*Ad*E	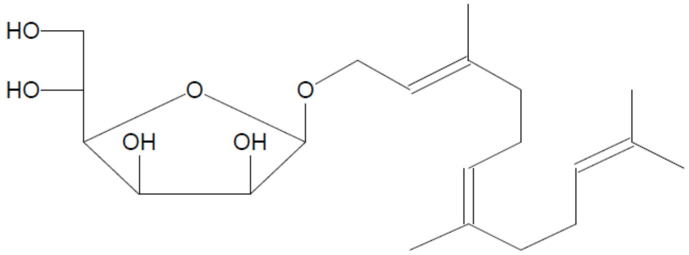

**Fenretinide**	C26H33NO2	53.432	655	713	6.97%	65646-68-6	ML	77816	391	*Ha*E	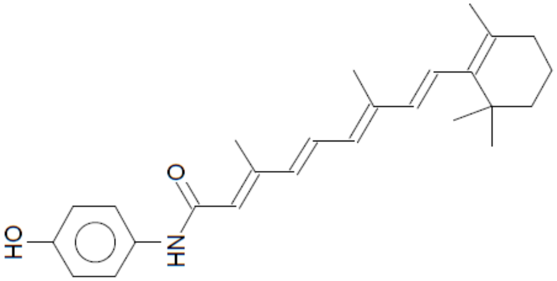
**Ursodeoxycholic acid**	C24H40O4	44.128	668	680	3.63%	128-13-2	ML	19201	392	*Ha*E	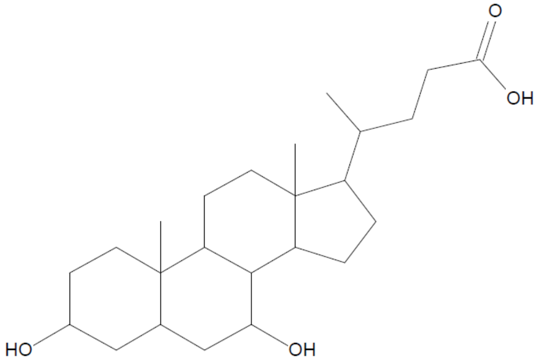
**3α,5α-Cyclo-ergosta-7,9(11),22t-triene-6β-ol**	$C_{28}H_{42}O$	29.846	596	651	4.07%	18978-72-6	ML	6910	394	*Pg*E	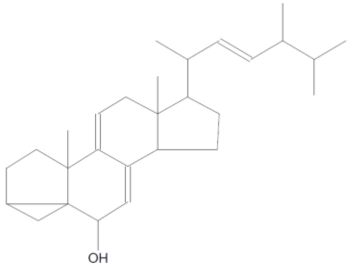
**3α,5α-Cyclo-ergosta-7,9(11),22t-triene-6β-ol**	$C_{28}H_{42}O$	30.354	611	672	7.27%	118978-72-6	ML	6910	394	*Pg*E	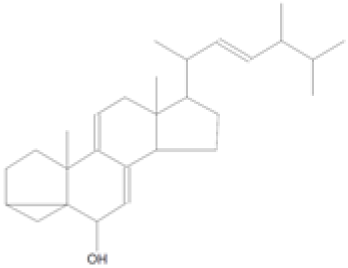

**3-Isopropyl-6a,7,10b-trimethyl-dodecahydro-benzo[f]chromene-7,8-dicarboxylic acid, dimethyl ester**	$C_{23}H_{38}O_5$	42.216	572	598	4.41%	—	ML	166394.	394	*Pg*E	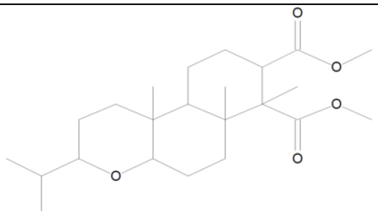
**1,2-Propanediol, 3-(hexadecyloxy)-, diacetate**	C23H44O5		696	709	6.13%	21994-82-1	ML	7437	400	*Ha*E	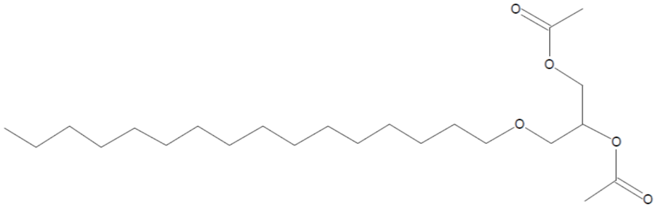
**Campesterol**	C28H48O	47.068	726	776	14.8%	474-62-4	ML	6713	400	*Ha*E	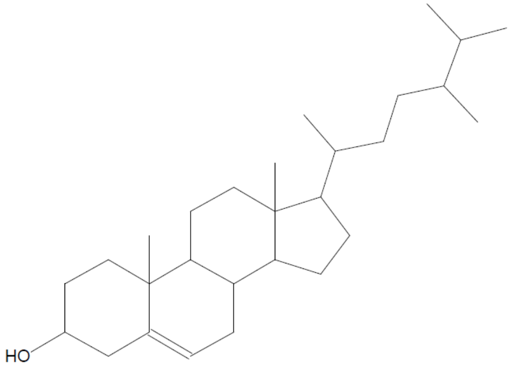
**5-Cholestene-3-ol, 24-methyl-**	C28H48O	47.068	705	751	6.29%	—	ML	6688	400	*Ha*E	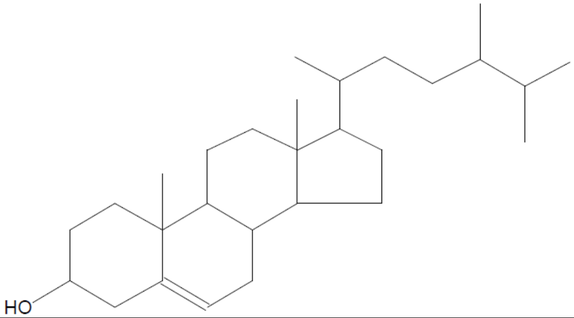

**Ergost-5-en-3-ol, (3β)-**	$C_{28}H_{48}O$	47.068	680	821	1.92%	4651-51-8	ML	6865	400	*Ha*E	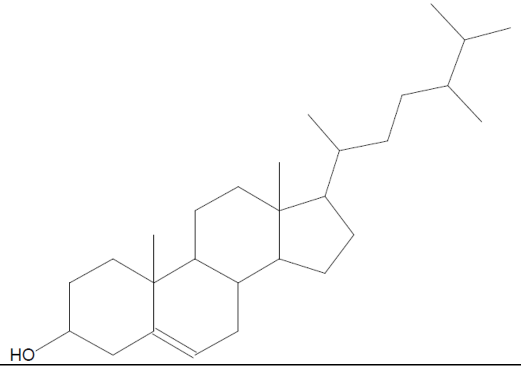
**cis-4,7,10,13,16,19-Docosahexaenoic acid, trimethylsilyl ester**	$C_{25}H_{40}O_2Si$	15.500	645	658	8.94%	—	ML	37920	400	*Ad*E	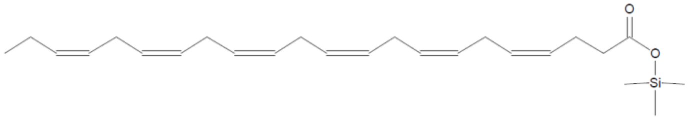
**25-Hydroxycholesterol**	$C_{27}H_{46}O_2$	40.316	664	683	5.61%	2140-46-7	ML	27474	402	*Ha*E	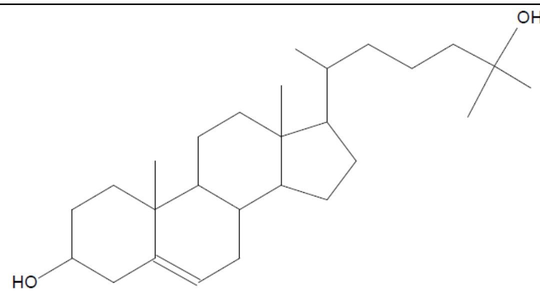
**25-Hydroxycholesterol**	$C_{27}H_{46}O_2$	44.128	670	703	3.94%	2140-46-7	ML	27474	402	*Ha*E	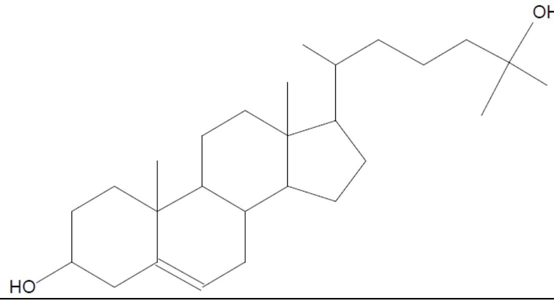

**Methyl 3β-acetoxy-24,23-dinor-5β-chol-5-enoate**	C25H38O4	15.725	620	656	4.54%	96092-29-4	ML	204501	402	*Ad*E	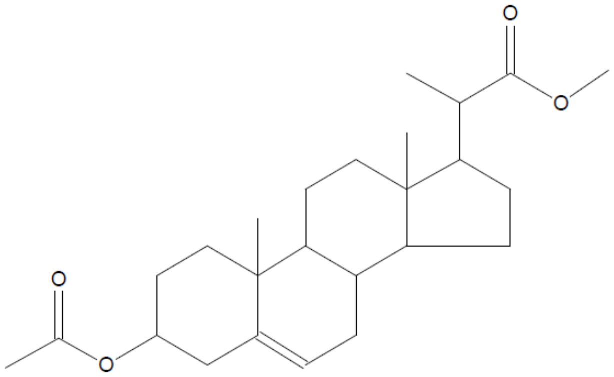
**5H-Cyclopropa[3,4]benz[1,2-e]azulen-5-one, 9-(acetyloxy)-1,1a,1b,4,4a,7a,7b,8,9,9a-decahydro-4a,7b,9a-trihyd**	C22H30O7	51.087	623	641	8.68%	70470-59-6	ML	147096	406	*Ha*E	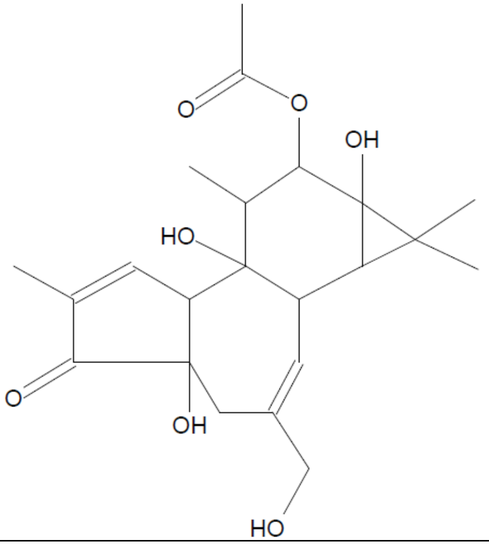

**Stigmasterol**	C29H48O	40.316	676	683	8.43%	83-48-7	RL	4902	412	*Ha*E	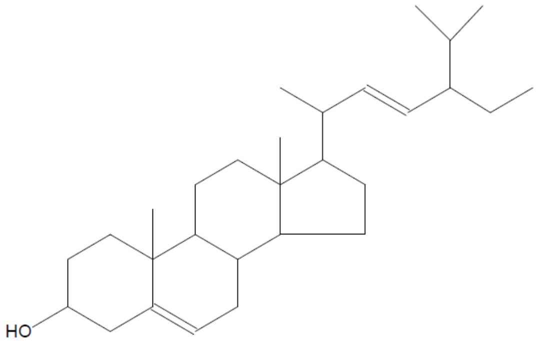
**Stigmasterol**	C29H48O	40.316	660	730	8.43%	83-48-7	ML	19475	412	*Ha*E	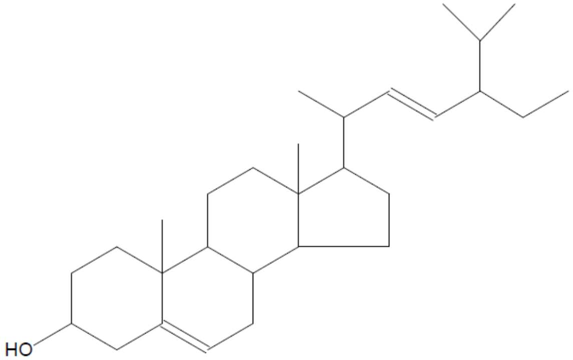
**Stigmasterol**	C29H48O	44.128	683	691	6.10%	83-48-7	RL	4902	412	*Ha*E	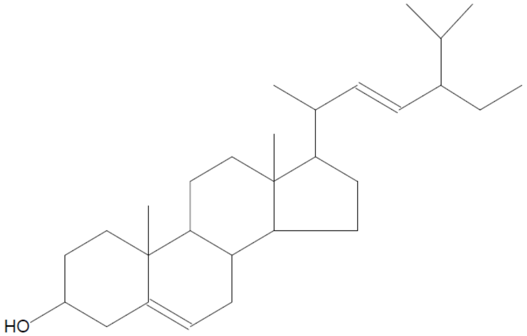

**β-Sitosterol**	C29H50O	47.068	743	777	27.6%	83-46-5	RL	1982	414	*Ha*E	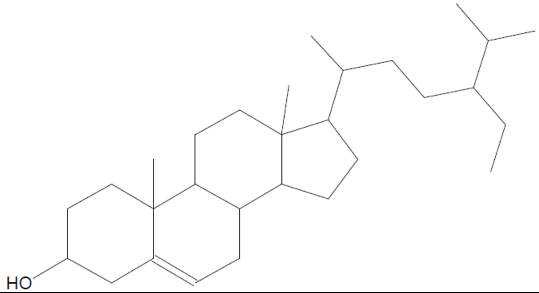
**γ-Sitosterol**	C29H50O	47.068	738	752	22.2%	83-47-6	ML	6839	414	*Ha*E	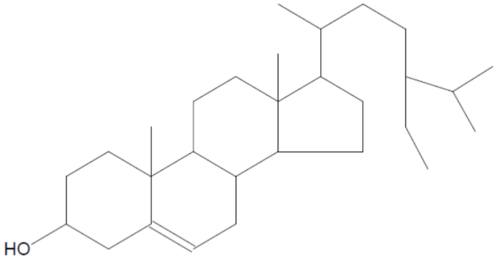
**9,10-Secocholesta-5,7,10(19)-triene-3,24,25-triol, (3β,5Z,7E)-**	C27H44O3	51.087	647	720	23.8%	40013-87-4	ML	6382	416	*Ha*E	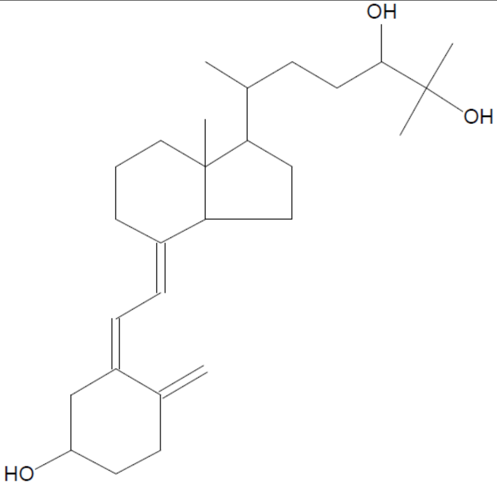

**Cyclobutane, 1,3-bis[2-(2-isopropyl-3,3-dimethyloxiran-2-yl)ethenyl]-2,4-diacetyl-**	C26H40O4	53.432	666	690	10.4%	70373-02-3	ML	11557	416	*Ha*E	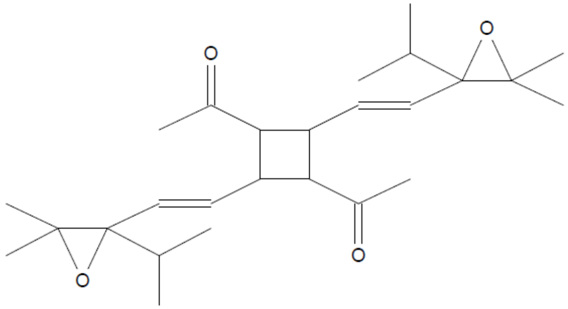
**10,12-Tricosadiynoic acid, trimethylsilyl ester**	C26H46O2Si	15.725	650	666	13.3%	—	ML	37660	418	*Ad*E	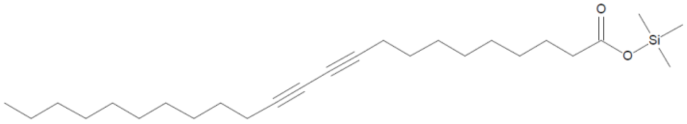
**8,14-Seco-3,19-epoxyandrostane-8,14-dione, 17-acetoxy-3β-methoxy-4,4-dimethyl-**	$C_{24}H_{36}O_6$	30.354	626	635	12.0%	—	ML	32504	420	*Pg*E	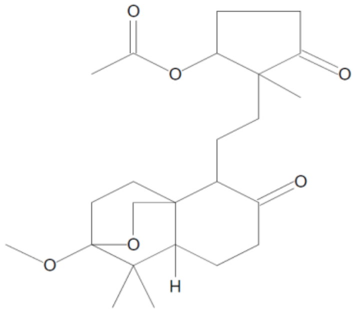
**Cholest-5-en-3-ol, 24-propylidene-, (3β)-**	C30H50O	44.128	693	731	8.64%	56362-45-9	ML	20346	426	*Ha*E	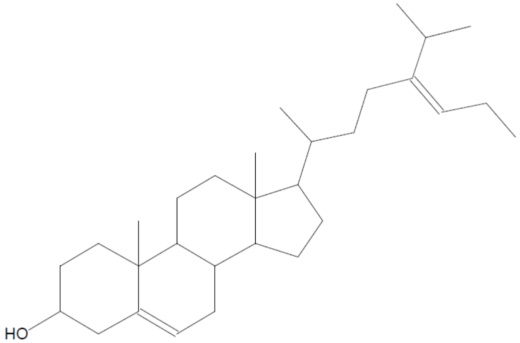

**1,2-Propanediol, 3-(octadecyloxy)-, diacetate**	C25H48O5		694	721	5.66%	21994-81-0	ML	7435	428	*Ha*E	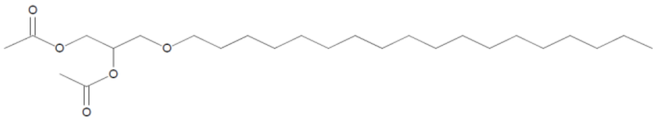
**Hexasiloxane, 1,1,3,3,5,5,7,7,9,9,11,11-dodecamethyl-**	$C_{12}H_{38}O_5Si_6$	42.216	593	700	9.12%	995-82-4	ML	39868	430	*Pg*E	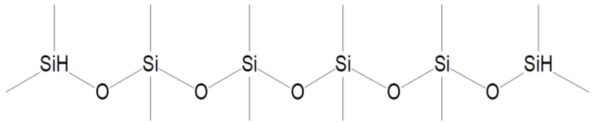
**Hexasiloxane, 1,1,3,3,5,5,7,7,9,9,11,11-dodecamethyl-**	$C_{12}H_{38}O_5Si_6$	46.889	590	681	8.13%	995-82-4	ML	39868	430	*Pg*E	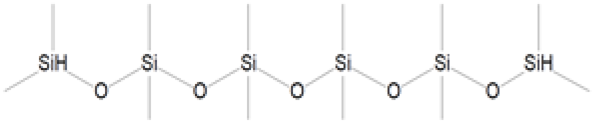
**Cholestan-3-one, cyclic 1,2-ethanediyl aetal, (5β)-**	C29H50O2	20.351	678	691	3.44%	25328-53-4	ML	65832	430	*Ad*E	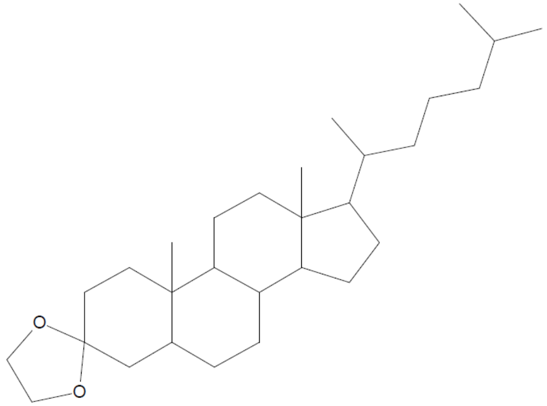
**i-Propyl 24-methyl-pentacos-5,9-dienoate**	C29H54O2	37.203	710	739	3.16%		ML	46210	434	*Ha*E	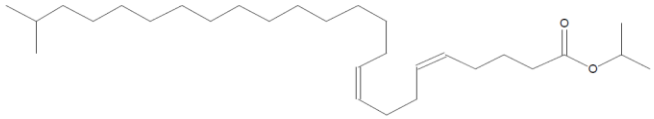

**Ethyl iso-allocholate**	C26H44O5	40.316	658	666	4.38%		ML	6654	436	*Ha*E	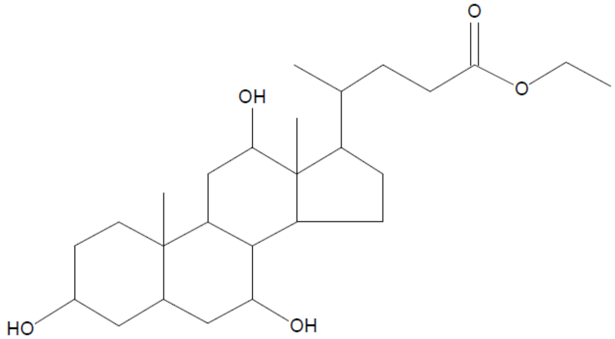
**Ergosta-5,22-dien-3-ol, acetate, (3β,22E)-**	C30H48O2	13.351	584	614	4.73%	2458-53-9	ML	5700	440	*Ad*E	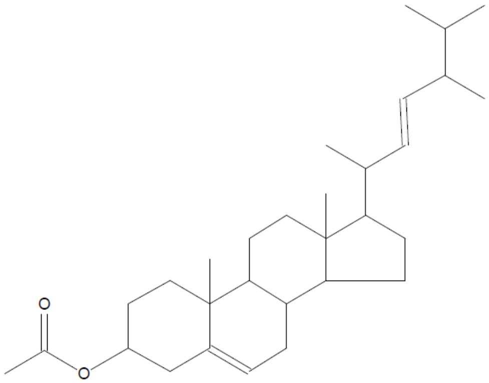
**Cholan-24-oic acid, 3-(acetyloxy)-7-oxo-, methyl ester, (3α,5β)-**	C27H42O5	53.432	646	684	4.75%	10452-65-0	ML	75683	446	*Ha*E	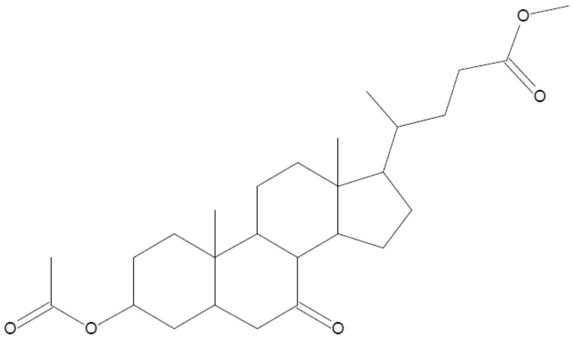

**Cholan-24-oic acid, 3-(acetyloxy)-7-oxo-, methyl ester, (3α,5β)-**	C27H42O5	56.828	617	644	5.63%	10452-65-0	ML	75683	446	*Ha*E	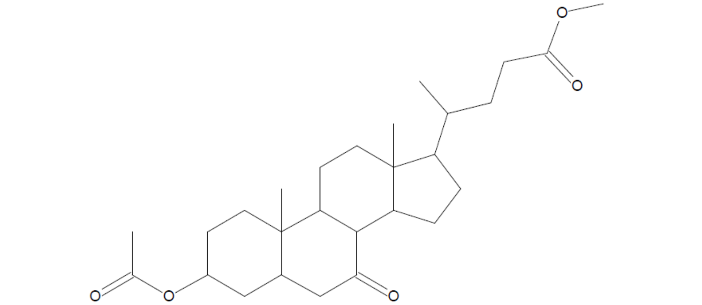
**Retinoyl-β-glucuronide 6',3'-lactone**	C26H34O7	51.087	614	654	6.30%	101470-87-5	ML	132135	458	*Ha*E	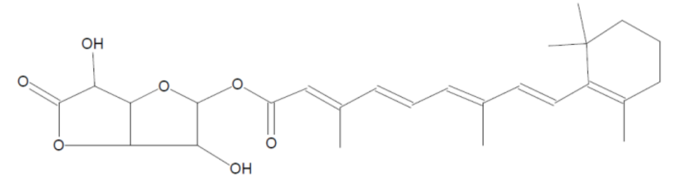
**13,14-Epoxyursan-3-ol, acetate**	C31H50O3	51.087	612	628	5.81%	—	ML	155588	470	*Ha*E	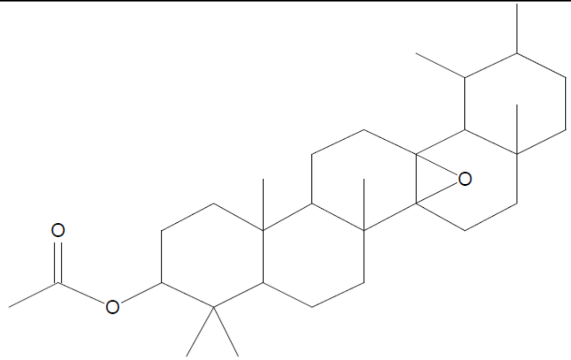
**2-Myristynoyl pantetheine**	C25H44N2 O5S	20.351	692	711	5.66%	—	ML	4307	484	*Ad*E	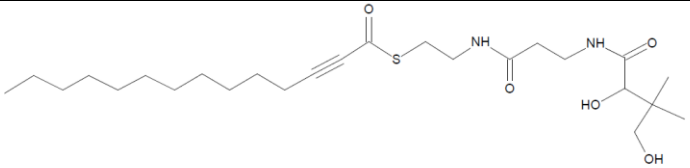
**9,10-Secocholesta-5,7,10(19)-triene-1,3-diol, 25-[(trimethylsilyl)oxy]-, (3β,5Z,7E)-**	C30H52O3S i	20.351	730	759	27.0%	55759-94-9	ML	18660	488	*Ad*E	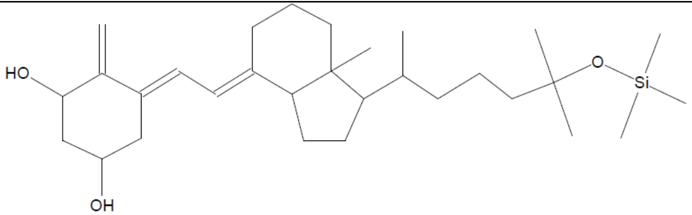

**17-Pentatriacontene**	$C_{35}H_{70}$	23.805	717	728	4.13%	6971-40-0	ML	22797	490	*Pg*E	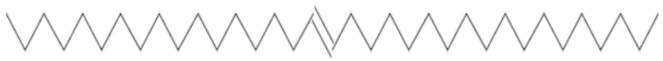
**Olean-12-ene-3,16,21,22,28-pentol, (3β,16α,21β,22α)-**	C30H50O5	56.828	614	664	4.97%	13844-01-4	ML	170277	490	*Ha*E	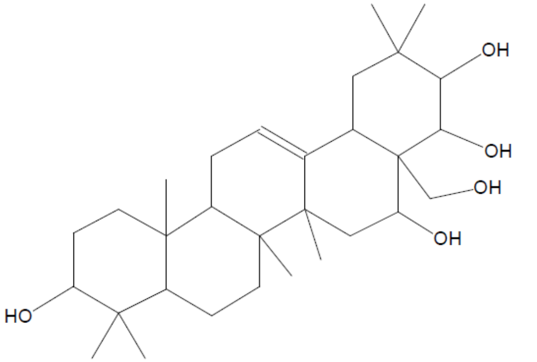
**9,12,15-Octadecatrienoic acid, 2,3-bis[(trimethylsilyl)oxy]propyl ester, (Z,Z,Z)-**	$C_{27}H_{52}O_4Si_2$	29.846	639	690	18.5%	55521-22-7	ML	41710	496	*Pg*E	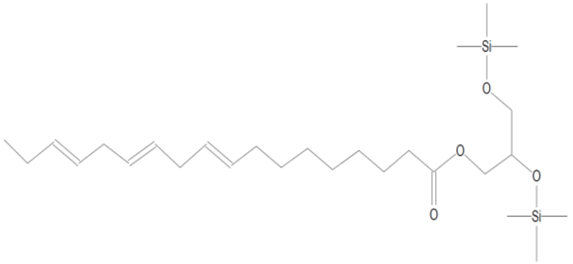
**9,12,15-Octadecatrienoic acid, 2,3-bis[(trimethylsilyl)oxy]propyl ester, (Z,Z,Z)-**	$C_{27}H_{52}O_4Si_2$	30.354	632	702	15.3%	55521-22-7	ML	41710	496	*Pg*E	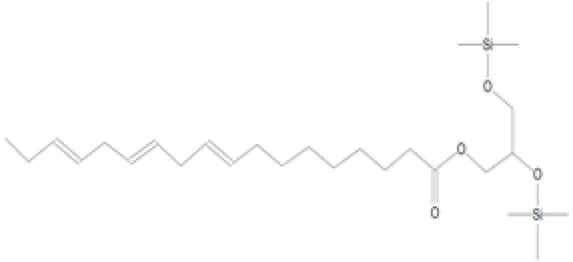

**9,12,15-Octadecatrienoic acid, 2,3-bis[(trimethylsilyl)oxy]propyl ester, (Z,Z,Z)-**	$C_{27}H_{52}O_4Si_2$	46.889	571	635	3.74%	55521-22-7	ML	41710	496	*Pg*E	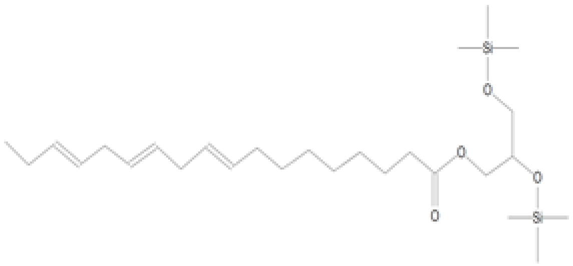
**9,12,15-Octadecatrienoic acid, 2,3-bis[(trimethylsilyl)oxy]propyl ester, (Z,Z,Z)-**	C27H52O4Si2	51.087	611	665	5.59%	55521-22-7	ML	41710	496	*Ha*E	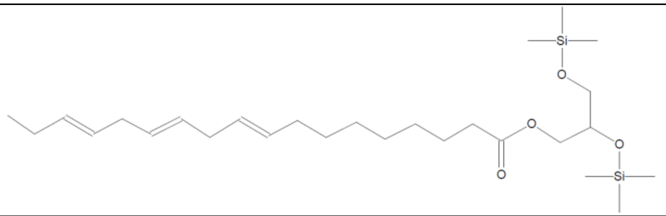
**9,12,15-Octadecatrienoic acid, 2,3-bis[(trimethylsilyl)oxy]propyl ester, (Z,Z,Z)-**	C27H52O4Si2	56.828	617	650	5.63%	55521-22-7	ML	41710	496	*Ha*E	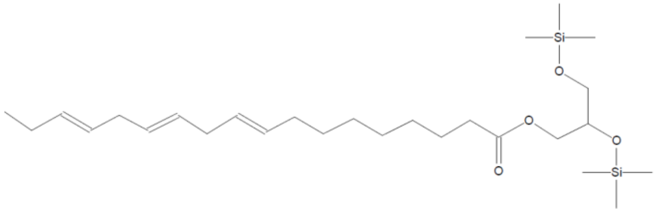
**1-Monolinoleoylglycerol trimethylsilyl ether**	$C_{27}H_{54}O_4Si_2$	29.846	656	702	33.9%	54284-45-6	ML	39425	498	*Pg*E	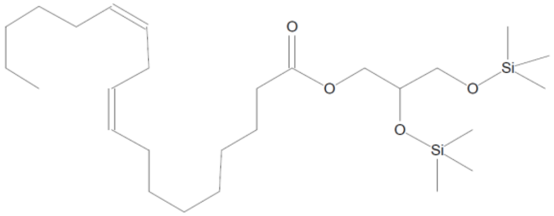
**1-Monolinoleoylglycerol trimethylsilyl ether**	$C_{27}H_{54}O_4Si_2$	30.354	645	711	23.7%	54284-45-6	ML	39425	498	*Pg*E	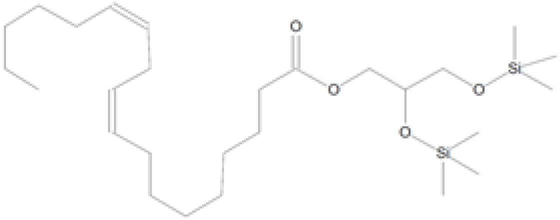

**1-Monolinoleoylglycerol trimethylsilyl ether**	$C_{27}H_{54}O_4Si_2$	42.216	609	670	15.8%	54284-45-6	ML	39425	498	*Pg*E	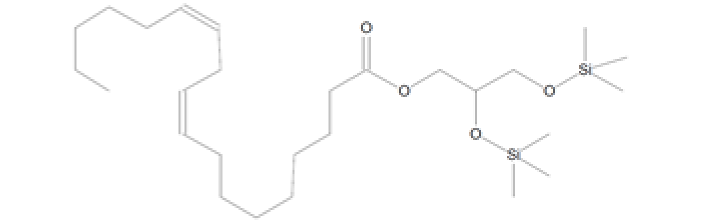
**1-Monolinoleoylglycerol trimethylsilyl ether**	$C_{27}H_{54}O_4Si_2$	46.889	612	670	20.6%	54284-45-6	ML	39425	498	*Pg*E	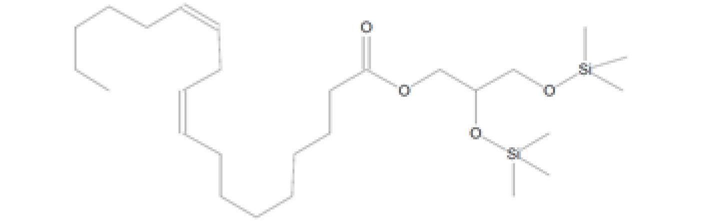
**1-Monolinoleoylglycerol trimethylsilyl ether**	C27H54O4Si2	51.087	625	679	9.41%	54284-45-6	ML	3942	498	*Ha*E	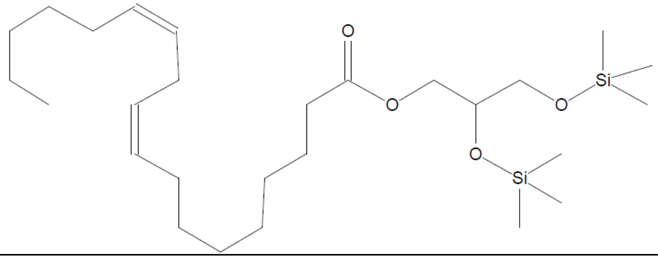
**1-Monolinoleoylglycerol trimethylsilyl ether**	C27H54O4Si2	56.828	625	661	7.65%	54284-45-6	ML	39425	498	*Ha*E	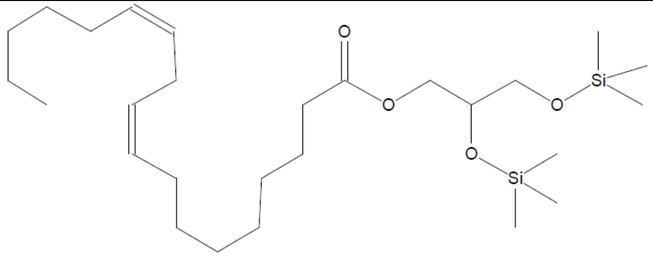
**Pseudosolasodine diacetate**	C31H49NO4	18.751	736	756	5.58%	—	ML	6693	499	*Ad*E	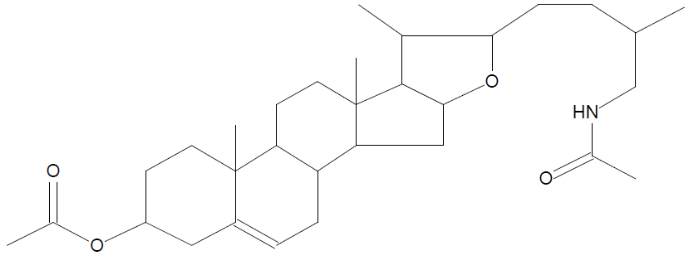

**Olean-12-ene-3,16,21,22,23,28-hexol, (3β,4α,16α,21β,22α)-**	C30H50O6	56.828	637	656	11.5%	13844-22-9	ML	170116	506	*Ha*E	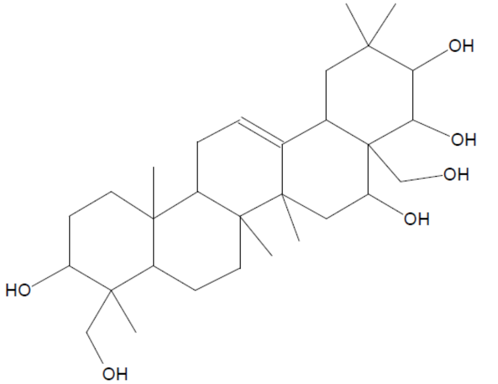
**10,12-Docosadiynedioic acid ditms**	C28H50O4Si2	15.725	636	649	7.79%		ML	37690	506	*Ad*E	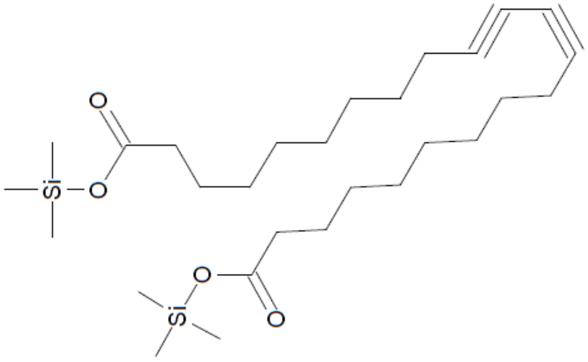
**Glycine, N-[(3α,5β)-24-oxo-3-[(trimethylsilyl)oxy]cholan-24-yl]-, methyl ester**	C30H53NO4Si	56.828	618	652	5.86%	57326-15-5	ML	102945	519	*Ha*E	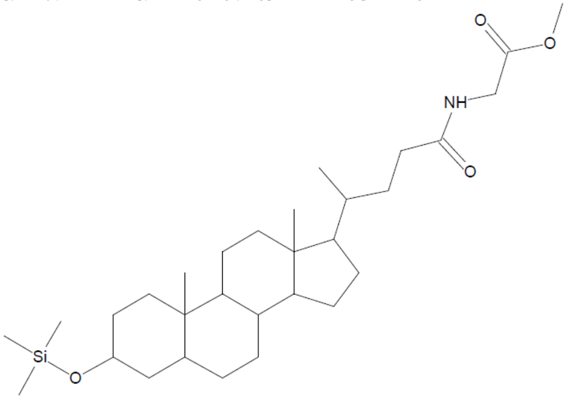

**9-Desoxo-9-x-acetoxy-3,8,12-tri-O-acetylingol**	C28H40O10	53.432	692	705	34.8%	—	ML	8015	536	*Ha*E	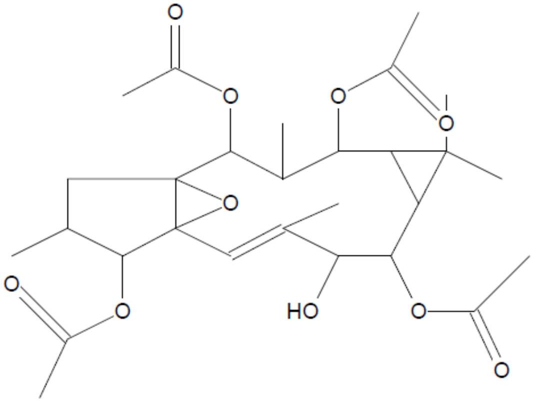
**Oleic acid, eicosyl ester**	C38H74O2	34.443	656	665	2.87%	22393-88-0	ML	25265	562	*Ha*E	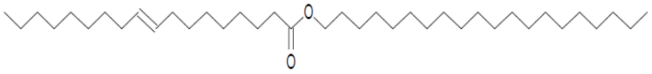
**Octasiloxane, 1,1,3,3,5,5,7,7,9,9,11,11,13,13,15,15-hexadecamethyl-**	$C_{16}H_{50}O_7Si_8$	42.216	581	692	6.07%	19095-24-0	ML	39429	578	*Pg*E	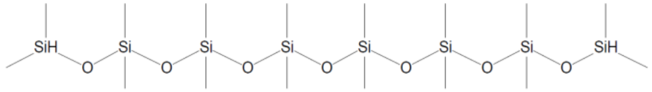
**Octasiloxane, 1,1,3,3,5,5,7,7,9,9,11,11,13,13,15,15-hexadecamethyl-**	$C_{16}H_{50}O_7Si_8$	46.889	587	680	7.18%	19095-24-0	ML	39429	578	*Pg*E	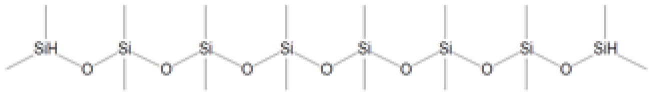
**Oleic acid, 3-(octadecyloxy)propyl ester**	C39H76O3	34.443	657	689	2.99%	17367-41-8	ML	22460	592	*Ha*E	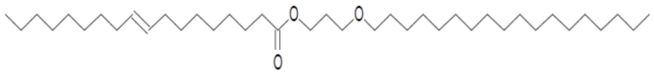
**Hexadecane, 1,1-bis(dodecyloxy)-**	$C_{40}H_{82}O_2$	23.805	727	758	6.27%	56554-64-4	ML	7351	594	*Pg*E	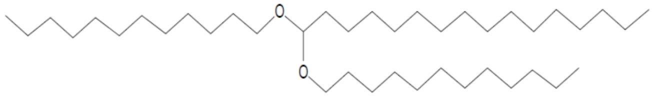

**.psi.,.psi.-Carotene, 1,1',2,2'-tetrahydro-1,1'-dimethoxy-**	C42H64O2	40.316	659	659	4.56%	13833-01-7	ML	37544	600	*Ha*E	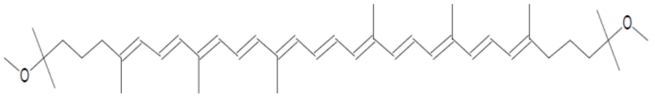
**.psi.,.psi.-Carotene, 1,1',2,2'-tetrahydro-1,1'-dimethoxy-**	C42H64O2	44.128	666	666	3.35%	13833-01-7	ML	37544	600	*Ha*E	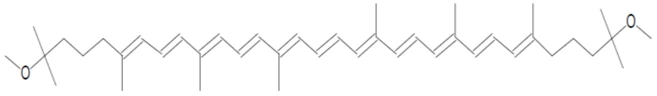
**Digitoxin**	C41H64O13	13.351	603	641	9.74%	71-63-6	RL	8938	764	*Ad*E	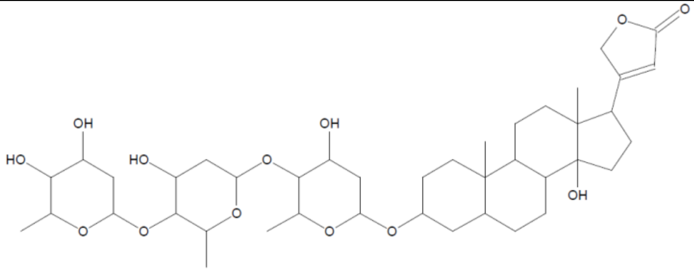
**Docosahexaenoic acid, 1,2,3-propanetriyl ester**	$C_{69}H_{98}O_6$	27.125	681	691	4.38%	11094-59-0	ML	44271	1022	*Pg*E	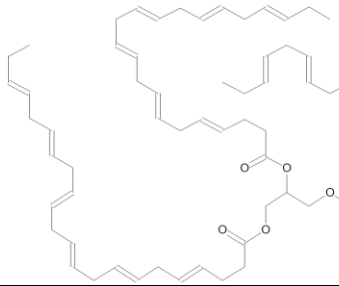

Abbreviations

MF: Match factor

RT: Retention time

RMF: Reverse match factor

Prob (%): Probability%

CAS#: Chemical abstract service registry number

Lib: Library

RL: Reliable library

ML: Main library

Ext: Extract
